# Metagenomic characterization of infected diabetic foot ulcers in North Africa: microbial diversity, virulome, and resistome profiling

**DOI:** 10.3389/fmicb.2026.1825173

**Published:** 2026-05-20

**Authors:** Muhanad Abdullah Abdulsamad, Sana Bardaa, Mouna Elleuch, Nour El Houda Mathlouthi, Mamdouh Ben Ali

**Affiliations:** 1 Laboratory of Microbial and Enzymatic Biotechnology and Biomolecules (LBMEB), Center of Biotechnology of Sfax (CBS), University of Sfax, Sfax, Tunisia; 2 Department of Biology, Faculty of Science, Sabratha University, Sabratha, Libya; 3 Department of Endocrinology, Hedi Chaker Hospital, Sfax University, Sfax, Tunisia; 4 Laboratoire de Recherche Toxicologie Microbiologie Environnementale et Santé (LR17ES06), Faculté des Sciences de Sfax, Université de Sfax, Sfax, Tunisia; 5 Astrum Biotech, Business incubator, Center of Biotechnology of Sfax (CBS), University of Sfax, Sfax, Tunisia

**Keywords:** antimicrobial resistance, biofilm, diabetic foot ulcers, microbiome, *Pseudomonas aeruginosa*, resistome, shotgun metagenomics, wound infection

## Abstract

This study provides the first shotgun metagenomic characterization of infected diabetic foot ulcers (DFUs) from North Africa. We analyzed two independent datasets with distinct roles: 25 non-infected US DFUs (PRJNA506988) served as an ecological reference cohort to characterize depth-stratified microbial community patterns and pre-infection ARG ecology; 15 infected Libyan DFUs constituted the primary characterization cohort. Metagenomic sequencing, taxonomic classification, resistome and virulome profiling, and metagenome-assembled genome (MAG) reconstruction were performed. In the US reference cohort, depth-dependent community shifts were documented: Fusobacteriota predominated in deeper ulcers, while Staphylococcaceae and Pseudomonadaceae were enriched in superficial wounds. Eighty ARGs were detected across depth groups, including *mecA* and the *mexAB-oprM* efflux system, in clinically non-infected wounds. In the Libyan cohort, four major opportunistic pathogens were identified: *Pseudomonas aeruginosa*, *Staphylococcus aureus*, *Acinetobacter baumannii*, and *Corynebacterium striatum*. From sample M13, a high-quality *P. aeruginosa* MAG (99.68% completeness, 0.89% contamination) was reconstructed, classified as ST664 and carrying 220 virulence factors, 60 antibiotic resistance genes (all confirmed by RGI v6.0.2), and 213 mobile genetic elements. These findings represent the first genomic evidence of ST664 in a North African DFU and underscore the need for metagenomics-guided antimicrobial stewardship in chronic wound management.

## 1 Introduction

Diabetic foot ulcers (DFUs) are a major arena of global health burden, affecting an estimated 6.3% of the adult population with diabetes worldwide (Zhang et al., 2017). This corresponds to about 33 million DFU-affected persons worldwide. Majorly responsible for morbidity, DFUs can lead to amputation of the lower limbs in about 20% of the affected population and precede 85% of amputations in diabetic patients (Zhang et al., 2017; McDermott et al., 2023). This further increases morbidity and the cost of healthcare while contributing to the risk of systemic infections, making chronic wounds one of the most difficult complications of diabetes to manage (Hicks et al., 2016). While great strides in the treatment of wounds and prevention of infection are being made, DFU management is still extremely complex mainly due to polymicrobial infections, emerging antibiotic-resistant strains, and the action of microbial biofilms in retarding healing (Doğruel et al., 2022; Kim et al., 2023). Traditionally, research has focused on the role of single pathogens, notably *Staphylococcus aureus* and *Pseudomonas aeruginosa*, in infections of DFU (Chai et al., 2021; Shalaby et al., 2023). Even so, more recent studies are indicating that the whole microbial community, instead of more isolated bacterial species, is crucial to wound progression and therapeutic response (Pouget et al., 2020). The DFU microbiome displays a huge degree of variability, with a depth-dependent microbial stratification, functional adaptations to hypoxia and inflammation (Gardner et al., 2013), and subsequently a fair chance of horizontal gene transfer (HGT) of antibiotic resistance determinants (Abe et al., 2020). While classical culture-based diagnostics remain the gold standard in clinical microbiology, most of the time, the cultured organisms miss out on major DFU-associated microbes, most notably anaerobes, biofilm-forming bacteria, and slow-growing pathogens, hence providing an incomplete picture of the wound infections and their resistance potential in clinical practice (Schmidt, 2022).

Recently, advances in shotgun metagenomics have facilitated the nonculturable characterization of microbial communities, providing strain resolution of pathogens, functional insights into microbial adaptations, and resistome characterization in totality (Mudrik-Zohar et al., 2022). Metagenomics departs from conventional techniques and simultaneously identifies all microorganisms within a wound, including the non-culturable ones, and provides a functional view of the virulence factors and antimicrobial resistance genes (ARGs) (Avila-Herrera et al., 2022; Kok et al., 2023; Be et al., 2014). Although most prior DFU microbiome studies have employed 16S rRNA amplicon sequencing, very few shotgun metagenomic datasets exist from regions outside North America and Western Europe, creating significant gaps in understanding regional microbiome variation and its clinical significance (Morsli et al., 2024). In this study, we aimed to fill this gap with the first shotgun metagenomic report on diabetic foot ulcers from North Africa. We used two independent datasets with distinct roles: publicly available metagenomic data from 25 non-infected US DFUs (PRJNA506988) served as an ecological reference cohort to characterize depth-stratified community patterns and pre-infection ARG ecology in non-infected wounds, providing the baseline against which our findings are contextualized; our prospective cohort of 15 infected Libyan DFUs constituted the primary characterization target of this study. These cohorts differ in infection status, geographic origin, and clinical context; this is explicitly acknowledged throughout the manuscript. All between-cohort observations are descriptive and hypothesis-generating.

## 2 Materials and methods

### 2.1 Impact of DFU depth on microbiome: a public data analysis

#### 2.1.1 Published DFU metagenomes of non-infected wounds

We accessed swab metagenomic data from project PRJNA506988 hosted by the European Nucleotide Archive (ENA). This dataset includes 25 swab samples obtained from diabetic foot ulcers (DFUs) in a cohort of individuals from the United States (Kalan et al., 2019). The samples are classified based on ulcer depth: 8 from surface DFUs (0 cm depth), 7 from superficial DFUs (depth > 0 to < 0.4 cm), and 10 from shallow DFUs (0.4 cm ≤ depth < 0.8 cm). All participants were diagnosed with diabetes (type I or type II) and met the inclusion criteria of being free from antibiotics for at least two weeks before sample collection and exhibiting no clinical signs of infection at the time of presentation. Participants continued other medications unrelated to DFU management and had no history of prior procedures (Supplementary Table S1).

#### 2.1.2 Taxonomic analysis

Taxonomic analysis was done using a comprehensive bioinformatics pipeline. Raw sequencing reads were processed, with the use of certain software such as Trimmomatic v0.39 (Bolger et al., 2014), to exclude probably contaminating sequences of adapter and low-quality bases for determining the true quality of DNA sequencing reads. Human reads were removed by alignment to the GRCh38 reference genome using HISAT2 v2.2.1 (Kim et al., 2015); unmapped reads were retained for downstream analysis. HISAT2 was selected for host depletion as it has been validated in published DFU metagenomics workflows and demonstrated equivalent host depletion efficiency to Bowtie2 for short paired-end reads (Kim et al., 2015). Taxonomic classification was performed on the sequenced reads with Kraken2 v2.1.3 with the PlusPF-16 database (downloaded November 2024; encompassing bacterial, viral, archaeal, fungal, and protozoan reference sequences) (Wood and Salzberg, 2014), followed by species-level abundance estimation with Bracken v3.0 (minimum threshold 10 reads) (Lu et al., 2017). Sequencing depth ranged from 62,791 to 10,724,064 raw reads per sample in the US cohort (median 415,972) and from 2,349,245 to 8,163,357 in the Libyan cohort (median 4,092,117). Full per-sample quality metrics are provided in Supplementary Table S2. Quality assessment of raw and host-depleted reads was performed with FastQC v0.11.7; aggregate reports are available in the Supplementary Table S2. To assess the influence of sequencing depth on diversity estimates, rarefaction analysis was performed by subsampling all US cohort samples to the minimum sample depth (*n* = 55,284 reads; sample 309) using the vegan package v2.6–4 (function rrarefy, 1,000 iterations, set.seed(42)). Shannon diversity indices were recalculated from rarefied data and compared to non-rarefied values. Rarefaction curves are provided in Supplementary Figure 1.

Shannon’s diversity index was used to assess alpha diversity, while one-way ANOVA was utilized to determine any statistically significant differences between groups. Bray–Curtis dissimilarity was used to calculate beta diversity, with statistical significance being determined by PERMANOVA within the vegan R library.

#### 2.1.3 Biostatistics and visualization

To make sure that the downstream analyses became more reliable, Pavian was set to create a comparative table of taxonomic reports between Kraken2 and Bracken, enabling a comprehensive and comparative evaluation of taxonomic profiles among the samples (Breitwieser and Salzberg, 2020). A series of filtering steps were employed next that were aimed at removing features of low quality probably arising from sequencing errors. Features with very low counts in just a few samples were simply considered to be artifacts owing to errors and were subsequently excluded. Taxonomic features underwent filtering on an applied threshold of at least 4 counts and were retained only when in at least 20% of the samples four or more counts were made. Features that showed little variation through the dataset were also subjected to assessment about exclusion, applying the filter to the range of the lowest 10 % of features through their interquartile range. To take into account variations in library sizes, CSS normalization was applied to correct the data. These filtering and normalization procedures were done through the MicrobiomeAnalyst platform.^1^ Statistical test selection was as follows. Prior to one-way ANOVA on Shannon diversity indices, normality was verified by Shapiro–Wilk test (Surface: *W* = 0.943, *p* = 0.713; Superficial: *W* = 0.931, *p* = 0.449; Shallow: *W* = 0.924, *p* = 0.399) and homogeneity of variance by Levene’s test (*F* = 2.340, *p* = 0.120); all parametric assumptions were met. Post-hoc pairwise comparisons used Tukey HSD correction. Kruskal-Wallis test was applied as a non-parametric confirmation. For beta diversity, PERMANOVA (adonis2 function, vegan R library, 999 permutations) was preceded by PERMDISP analysis to confirm homogeneous multivariate dispersion within groups, ensuring that PERMANOVA results reflect community composition differences rather than dispersion differences. CSS normalization (Paulson et al., 2013) was selected over rarefaction to avoid discarding data from low-depth samples; as a sensitivity analysis, all edgeR differential abundance results were reproduced with TMM normalization and conclusions were qualitatively identical. All reported *p*-values for differential abundance analyses (edgeR) were corrected for multiple testing by the Benjamini-Hochberg procedure (FDR ≤ 0.05). Complete statistical results are provided in Supplementary Table S2. The EdgeR (Robinson et al., 2010) approach was used for the differential abundance analysis with a cutoff value of ≤0.05 for statistical significance. Data visualization was done using a range of R packages to demonstrate clear and well-informed graphical representations. Detailed and well-informed plots were prepared with the help of ggplot2 (Wickham, 2011) while dplyr was responsible for data manipulation and processing.

#### 2.1.4 Pathogenicity analysis

Metagenomic data were assembled using Shovill^2^ with the MEGAHIT (Li et al., 2015) assembler to generate high-quality draft assemblies. These assemblies were analyzed for antibiotic resistance gene detection using the Abricate tool^3^ with the CARD database (Alcock et al., 2023) used as a resistance screening reference. Virulence factors were identified from the VFDB database (Liu et al., 2022), The functional pathways and biological processes to which the factors were attached were further annotated with UniProt (UniProt Consortium, 2015). A Venn diagram was generated to illustrate the differences in pathogenicity profiles between the groups in terms of resistance and virulence features.^4^

### 2.2 Microbial infections in DFU: a Libyan cohort study

#### 2.2.1 Subjects and ethical approval

A prospective cohort study was conducted to investigate DFU microbiota in a Libyan population. Fifteen participants with DFU were recruited for this study. The study was approved by the Institutional Review Board approval at National cancer institute (NCI) and written informed consent was obtained from all participants.

#### 2.2.2 Patient selection and sample collection

Patients were recruited from Sabratha Teaching Hospital Libya according to the following criteria: patients with a confirmed diagnosis of diabetes, presenting an active DFU at the time of enrollment and showing clinical signs of local infection such as erythema and purulent discharge. Patients with previous antibiotic intake before sampling and those having systemic infections other than DFU were excluded from the study. Clinical parameters such as HbA1c, blood glucose, CRP, body temperature, partial amputation history, and ulcer characteristics including purulent secretion, were recorded for each participant (Supplemental Data S1). Deep tissue ulcer specimens were obtained by sterile swabbing following debridement and cleaning the ulcer area with sterile saline. Swabs were stored immediately at −80 °C until DNA extraction.

#### 2.2.3 DNA extraction, library preparation, sequencing

Swab samples were subjected to DNA extraction by the DNeasy Blood and Tissue Kit according to the manufacturer’s instructions (QIAGEN). Before the DNA extraction process, swabs were incubated for 30 min in phosphate-buffered saline solution pre-warmed to 37 °C. Later, the sample was centrifuged at 3500 RPM for 5 min. Then, the supernatant was discarded, the precipitate resuspended in the lysozyme solution and incubated at 37 °C for 30 min. Subsequently, DNA quality and purity were measured using a QIAxpert system from QIAGEN by checking the A260/A280 and A260/A230 ratios. DNA samples were previously normalized to 10 ng/μL and then used for the preparation of the sequencing library with the QIAseq FX DNA Library Kit from QIAGEN according to the manufacturer’s recommendations. The concentrations were quantified in a QUBIT (Thermo Fisher Scientific), and QIAxcel Connect (QIAGEN) capillary electrophoresis was employed for determining size distribution. These latter steps normalized libraries to 4 nM, followed by their pooling in readiness for sequencing on the Illumina NextSeq 550, set with High Output kit configuration in a paired-end mode with read lengths of 2x150bp.

#### 2.2.4 Metagenome-assembled genome (MAG) recovery and refinement

Taxonomy and pathogenicity analyses, as done in earlier sections (1.2 and 1.4), were conducted before MAG recovery. First, contigs were assembled using MEGAHIT and then binned with the metaWRAP (Uritskiy et al., 2018) pipeline, which includes a multi-algorithm method of binning, including MaxBin2 (Wu et al., 2016), MetaBAT2 (Kang et al., 2019), and CONCOCT (Alneberg et al., 2014) that leveraged tetranucleotide frequency, GC content, and coverage information. To retain only high-quality bins following the MIMAG (Bowers et al., 2017) standards, the ‘bin_refinement’ module in metaWRAP was applied with parameters -c 90 and -x 5, thus assuring at least 90% completeness and less than 5% contamination. Further refinement was done with MAGpurify (Nayfach et al., 2019), and then the quality of the bins was checked with CheckM (Parks et al., 2015). To minimize false-positive ARG detection from assembly artifacts, ARG inclusion required confirmation by three independent methods: (i) Prokka v1.14.6 genome annotation (Matsen et al., 2010); (ii) ABRicate v1.0.1 against the CARD database (Alcock et al., 2023; Li et al., 2015); identity ≥70%, coverage ≥80%, minimum contig length 500 bp, minimum contig coverage 10×; and (iii) read-based validation using RGI v6.0.2 (Resistance Gene Identifier; protein homolog model; strict bitscore cutoff ≥900) applied to unmapped reads. Only ARGs confirmed by all three methods were reported.

#### 2.2.5 Taxonomy, annotation, and phylogeny of MAGs

The GTDB (Chaumeil et al., 2020) toolkit used both FastANI (Jain et al., 2018) and pplacer (Matsen et al., 2010) for the taxonomic classification of MAGs. Prokka (Seemann, 2014) was used for the annotation, whereas the ARGs were identified using CARD (Alcock et al., 2023). MobileOG-DB (Brown et al., 2022) was used for the detection of mobile genetic elements, and Alien Hunter (Vernikos and Parkhill, 2006) for horizontal gene transfer prediction. Besides, MLST (Maiden et al., 2013)/rMLST (Jolley et al., 2012) analyses had been performed. The phylogenetic distances have been calculated via Mash/MinHash (Ondov et al., 2016), and sequences were then aligned using MAFFT (Katoh et al., 2002). iTOL (Letunic and Bork, 2021) was used to visualize the RAxML-generated phylogenetic tree (Stamatakis, 2014). The MAG was then compared against reference genomes via BLAST and coverage was depicted in a circular genome plot prepared with Circos (Krzywinski et al., 2009).

## 3 Results

### 3.1 Clinical characteristics

The mean age was 52.3 ± 15.2. Ages in the cohort ranged from 24 to 85 years, with 60% (*n* = 9) being male and 40% (*n* = 6) being female. All participants had a confirmed diagnosis of diabetes mellitus (Type I: *n* = 1; Type II: *n* = 14). The durations of diabetes ranged from 8 months to 35 years. Of the 15 participants, 8 had previously experienced partial amputation, involving fingers (*n* = 6) and part of the foot (*n* = 2), while 7 had never been amputated. In the first part of the study, publicly available metagenomic data from the PRJNA506988 project involving 25 swab samples from DFU in a U. S. cohort were analyzed. The total file size of the dataset was 8.03 GB with an average of 0.32 GB per sample. Cohort characteristics included 55 years mean age and 19.23% were female. All patients were diagnosed with diabetes, including both type I and type II. They were clinically non-infected, received no antibiotics, and had no history of amputations when the samples were taken (Supplementary Table S1).

The metagenomic analysis of the 25 diabetic foot ulcers (DFUs) yielded 30,386,465 reads in total. Of these, 77.64% (or 23,593,211) reads were classified, with 98.79% (or 23,308,042) reads identified as microbial reads. The bacterial sequences dominated the microbial fraction, as 99.78% (23,258,119) were made up of microbial reads, while viral sequences were 0.19% (45,339). A mean of 12.7% of reads per sample remained unclassified by Kraken2 in the US cohort (median 9.7%, range 1.0–37.1%; Supplementary Table S2; Supplementary Figure 2). The two samples with the highest unclassified rates (sample 139: 37.1%; sample 147: 33.9%) were among the most deeply sequenced (1.32 M and 10.7 M reads, respectively), suggesting these reads represent genuine novel or undercharacterized wound-associated taxa not covered by the PlusPF-16 database, rather than sequencing artifacts. In the Libyan cohort, unclassified read rates were consistently low (mean 0.96%, range 0.6–2.1%), consistent with the microbial fraction being dominated by well-characterized reference species. Host DNA content in Libyan wound swab samples ranged from 36.4 to 98.5% (mean 87.2%; see Supplementary Table S2; Supplementary Figure 3). This is consistent with published values for DFU swab metagenomics without prior physical host depletion ((Kalan et al., 2019): 60–80%; (Mudrik-Zohar et al., 2022): 55–90%). Wound swabs inherently co-collect human material including wound exudate, sloughed epithelial cells, neutrophils, macrophages, and erythrocytes (Mudrik-Zohar et al., 2022; Kalan et al., 2019). The variation in microbial fraction across Libyan samples reflects inter-patient differences in bacterial burden at time of swabbing, a biologically meaningful finding. Sample M13, with the highest microbial fraction (63.6%), harbored a dominant *P. aeruginosa* infection; samples M12 and M22 (98.5% human DNA each) represent patients with low bacterial colonization at sampling. Full per-sample metrics are provided in Supplementary Table S2 and Supplementary Figure 3.

**Figure 1:**
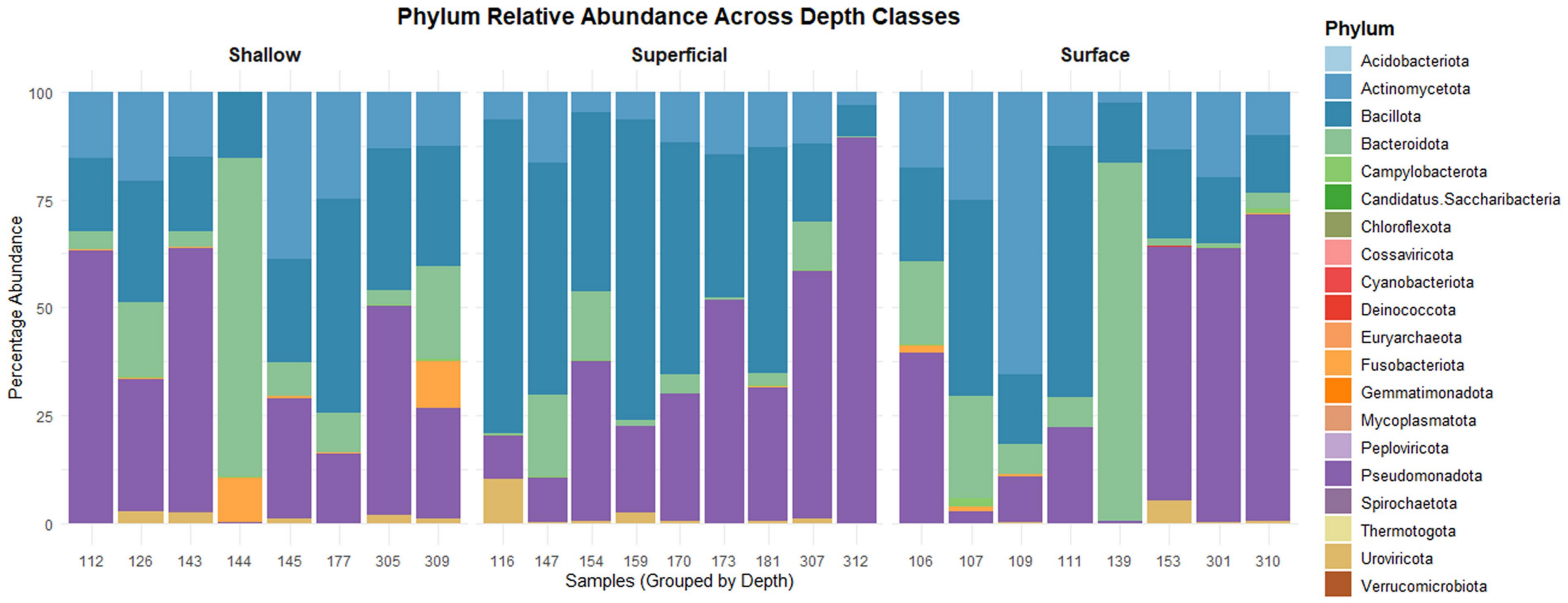
Phylym relative abundance across depth classes.

The results of alpha and beta diversity observed were not significantly different between ulcer depths and so suggested that a similar complexity of communities was present in the various depths under examination (Supplementary Table S3). The main phyla that were prevalent in the DFU microbiome include Pseudomonadota, Bacillota, Actinomycetota, and Bacteroidota, along with others such as Fusobacteriota, Campylobacterota, and Spirochaetota, which form part of the low-abundance phyla that also further confirms these wounds’ diverse microbial ecology (Supplementary Table S3; Figure 1).

### 3.2 Differential abundance across ulcer depths

Beta diversity analysis showed that microbial community composition clustered by ulcer depth (PERMANOVA Superficial vs. Shallow: R^2^ = 0.112, *p* = 0.047; all other depth comparisons: *p* > 0.05). PERMDISP analysis confirmed homogeneous dispersion within groups (all *p* > 0.2), indicating that the borderline PERMANOVA result reflects genuine community differences rather than dispersion artifacts. Alpha diversity (Shannon H′) did not differ significantly across depth groups (one-way ANOVA: *F* = 2.140, *p* = 0.141; Kruskal-Wallis: H = 4.127, *p* = 0.127; post-rarefaction ANOVA: *F* = 2.310, *p* = 0.130), indicating similar overall community richness across depths (Supplementary Table S2). This is consistent with depth-specific taxonomic shifts occurring without an overall change in community complexity. Complete PERMANOVA statistics are provided in Supplementary Table S2.

Comparison between ulcer depths revealed distinct phylum-level patterns (Supplementary Figure 4). Fusobacteriota showed a significantly higher positive correlation with shallow ulcers rather than superficial ulcers (*p* < 0.001), whereas Bacillota also showed a significant positive correlation with superficial ulcers rather than with shallow ulcers (*p* = 0.0027). Bacteroidota (*p* = 0.0012), Spirochaetota (*p* = 0.0071), and Campylobacterota (*p* = 0.019) correspondingly represented positive coefficients when comparing surface to superficial; whereas Bacillota retained its positive relationship when comparing superficial to the surface for ulcers (*p* = 0.014) (Supplementary Tables S3, S4; Table 1).

**Table 1:** Comparative analysis of microbial abundance by depth using edgeR.

		Depth	*p*-value	FDR
Phylum	Fusobacteriota	Shallow > Superficial	0.00012691	0.0011422
	Bacillota	Superficial > Shallow	0.012302	0.012302
		Superficial > Surface	0.014387	0.042353
	Spirochaetota	Surface > Superficial	0.0071048	0.031972
	Campylobacterota	Surface > Superficial	0.018823	0.042353
Family	Prevotellaceae	Shallow > Superficial	5.6422e-05	0.0012461
		Shallow > Surface	0.000576	0.008064
	Veillonellaceae	Superficial > Shallow	0.0045798	0.029774
	Staphylococcaceae	Superficial > Shallow	0.0059592	0.032088
	Pseudomonadaceae	Superficial > Shallow	0.0084291	0.042145
		Superficial > Surface	0.003159	0.036855
	Enterococcaceae	Superficial > Surface	0.0042219	0.042219
	Neisseriaceae	Surface > Superficial	0.00086827	0.030389
Genus	Haemophilus	Superficial > Shallow	0.0096693	0.037914
	Prevotella	Shallow > Superficial	8.3986e-05	0.0041713
		Shallow > Surface	0.0097457	0.0097457
	Pseudomonas	Superficial > Shallow	0.0031439	0.020664
	Staphylococcus	Superficial > Shallow	0.0020018	0.014991
Species	*Prevotella melaninogenica*	Shallow > Superficial	5.0202e-05	0.002667
		Shallow > Surface	0.00022814	0.010773
	*Pseudomonas aeruginosa*	Superficial > Shallow	0.0015574	0.016547
		Superficial > Surface	4.8279e-05	0.0041037
	*Rothia mucilaginosa*	Superficial > Shallow	0.0034241	0.029699
	*Staphylococcus aureus*	Superficial > Shallow	0.005758	0.043699
	*Streptococcus anginosus*	Shallow > Superficial	0.0038172	0.031229
		Surface > Superficial	0.0036308	0.035084
	*Streptococcus oralis*	Shallow > Superficial	0.0038393	0.031229
		Shallow > Surface	0.0032516	0.03071
	*Streptococcus mitis*	Superficial > Surface	0.0014356	0.025095
	*Staphylococcus epidermidis*	Superficial > Surface	0.0036322	0.035084

At the family level, the shallower ulcers had different microbial profiles than the superficial ones. Out of all the bacterial families, Prevotellaceae was found to have a positive correlation with shallow ulcers than superficial ulcers (*p* < 0.001) while Veillonellaceae (*p* = 0.0046), Staphylococcaceae (*p* = 0.0060), and Pseudomonadaceae (*p* = 0.0084) were found in higher quantities in superficial ulcers as compared to shallow ulcers. Neisseriaceae was found to have a positive correlation with surface ulcers as compared to superficial ulcers (*p* < 0.001) while Pseudomonadaceae (*p* = 0.0032) and Enterococcaceae (*p* = 0.0042) were found in higher quantities in superficial ulcers as compared to surface ulcers. Interestingly, Prevotellaceae were found in higher quantities in shallow ulcers as compared to surface ulcers (*p* < 0.001) (Supplementary Tables S3, S4; Supplementary Figure 5; Table 1).

The analysis showed a significant genus-level differentiation of ulcer depth. In the shallow vs. superficial analysis, Prevotella (*p* < 0.001) and Neisseria (*p* = 0.0074) were found to be more prevalent in shallow ulcers, while Staphylococcus (*p* = 0.0020), Pseudomonas (*p* = 0.0031), and Haemophilus (*p* = 0.0097) were more prevalent in superficial ulcers than in shallow ulcers. The results also showed that Prevotella was more abundant in shallow ulcers than surface ulcers (*p* < 0.001) (Supplementary Tables S3, S4; Supplementary Figure 6; Table 1). The most significant species-level differences were noted between shallow and superficial ulcers. *Prevotella melaninogenica* (*p* < 0.001), *Streptococcus anginosus* (*p* = 0.0038), and *S. oralis* (*p* = 0.0038) were positively correlated with shallow ulcers when compared to superficial ulcers. In contrast, *Pseudomonas aeruginosa* (*p* = 0.0016), *Rothia mucilaginosa* (*p* = 0.0034), and *Staphylococcus aureus* (*p* = 0.0058) were found to be more prevalent in superficial ulcers than in shallow ones (Supplementary Tables S3, S4; Table 1). In comparisons between surface and shallow ulcers, *P. melaninogenica* and *S. oralis* exhibited significantly higher levels in shallow ulcers (*p* < 0.001 and *p* = 0.0033, respectively). When looking at surface versus superficial ulcers, *S. anginosus* was positively correlated with surface ulcers (*p* = 0.0036), while *P. aeruginosa* (*p* < 0.001), *S. epidermidis* (*p* = 0.0036), and *S. mitis* (*p* = 0.0014) were more abundant in superficial ulcers compared to surface ulcers (Supplementary Figure 7).

### 3.3 Antibiotic resistance gene distribution

The metagenomic analysis showed that 80 ARGs were present across all samples; however, their pattern of distribution associated with ulcer depth differed (Supplementary Table S5). Only five ARGs appeared in ulcers of all depths: tetM (to resist tetracycline), SAT-4 (to fight off streptothricin) CfxA3 (to counter beta-lactams), APH(3′)-IIIa (to fend off aminoglycosides), and ErmA (to neutralize macrolides). All ARG annotations in this section represent genomic sequences with verified homology (BLAST identity ≥70%, coverage ≥80%) to experimentally characterized resistance determinants in the CARD database (Li et al., 2015). Their detection indicates genomic resistance potential but does not confirm active gene expression, functional resistance protein production, or clinical treatment failure.

The shallow and superficial ulcers harbored the highest number, 38, of shared ARGs coding for a variety of multidrug efflux pump proteins of the Mex family, namely, mexA, mexB, mexC, mexD, mexE, mexF, mexH, mexI, mexJ, mexL, mexM, mexN, mexP, mexQ, mexX, and mexY, along with their associated outer membrane proteins, namely, OprJ, OprM, opmD, opmE, and opmH. Equally, there were shared genes that confer resistance on specific classes of antibiotics: fosA confers resistance to fosfomycin, APH(3′)-IIb to aminoglycosides, and OXA-486 to beta-lactams (Supplementary Table S5). Superficial and surface ulcers share five common ARGs: tetQ (tetracycline resistance), mel (macrolide resistance), ANT(4′)-Ib (aminoglycoside resistance), and two erythromycin resistance genes (ErmF and ErmX). We detected three ARGs exclusively in shallow ulcers: tet(33) (tetracycline resistance), PC1_beta-lactamase_(blaZ) (beta-lactam resistance), and lmrP (multidrug resistance) (Supplementary Table S5). The superficial ulcers had the most unique ARGs with 25, in which genes coding for methicillin resistance (mecA), fluoroquinolone resistance (norA), and numerous regulatory genes (arlR, arlS, mgrA, and marA) were found. Special attention was given to genes encoding resistance against multiple classes of antibiotics: dfrK and dfrC, encoding trimethoprim resistance; mphC, encoding macrolide resistance; and lnuA, encoding lincosamide resistance. Surface ulcers contained four unique ARGs: cmx- chloramphenicol resistance, two aminoglycoside resistance genes APH(6)-Id, APH(3″)-Ib, and cepA_beta-lactamase resistance to beta-lactams (Supplementary Table S5).

### 3.4 Virulence factors and functional profiles across diabetic foot ulcer depths

**Figure 2:**
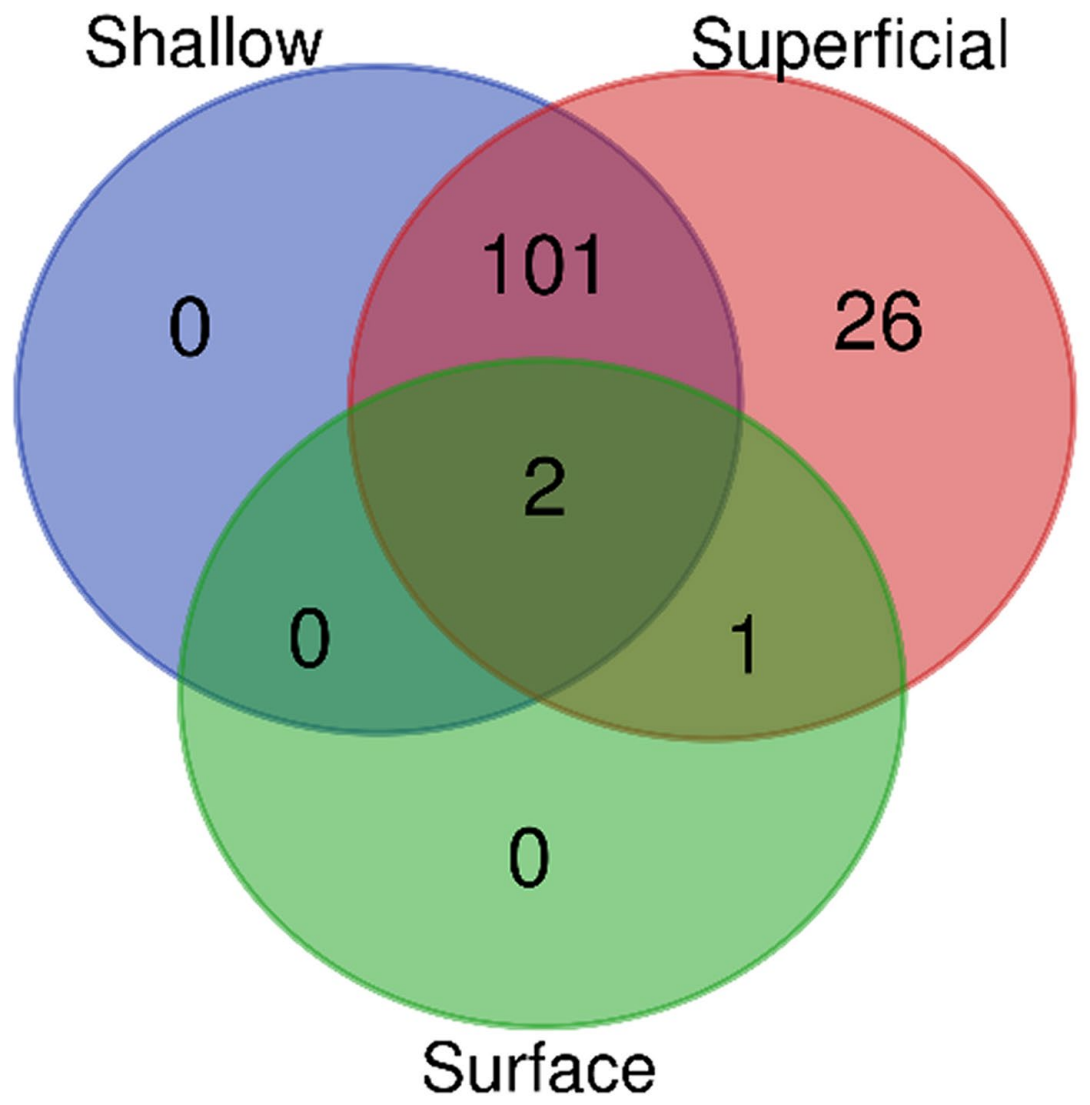
Distribution of virulence factor-associated biological processes by depth.

The virulence factor and functional analysis were performed on bacterial isolates from diabetic foot ulcers categorized into three depths surface, superficial, and shallow. Marked variations in the type of virulence factors, species of bacteria, biological processes, and metabolic pathways were observed in different depths (Supplementary Table S5). In the surface ulcer group, there were only three virulence factors associated with *Staphylococcus aureus*: non-specific protein-tyrosine kinase, gamma-hemolysin component A (H-gamma-2), and ESAT-6 secretion machinery protein EssA. Correspondingly, only two biological processes were detected in surface ulcers: phosphorylation and extracellular polysaccharide biosynthetic process (Supplementary Table S5; Figure 2).

**Figure 3:**
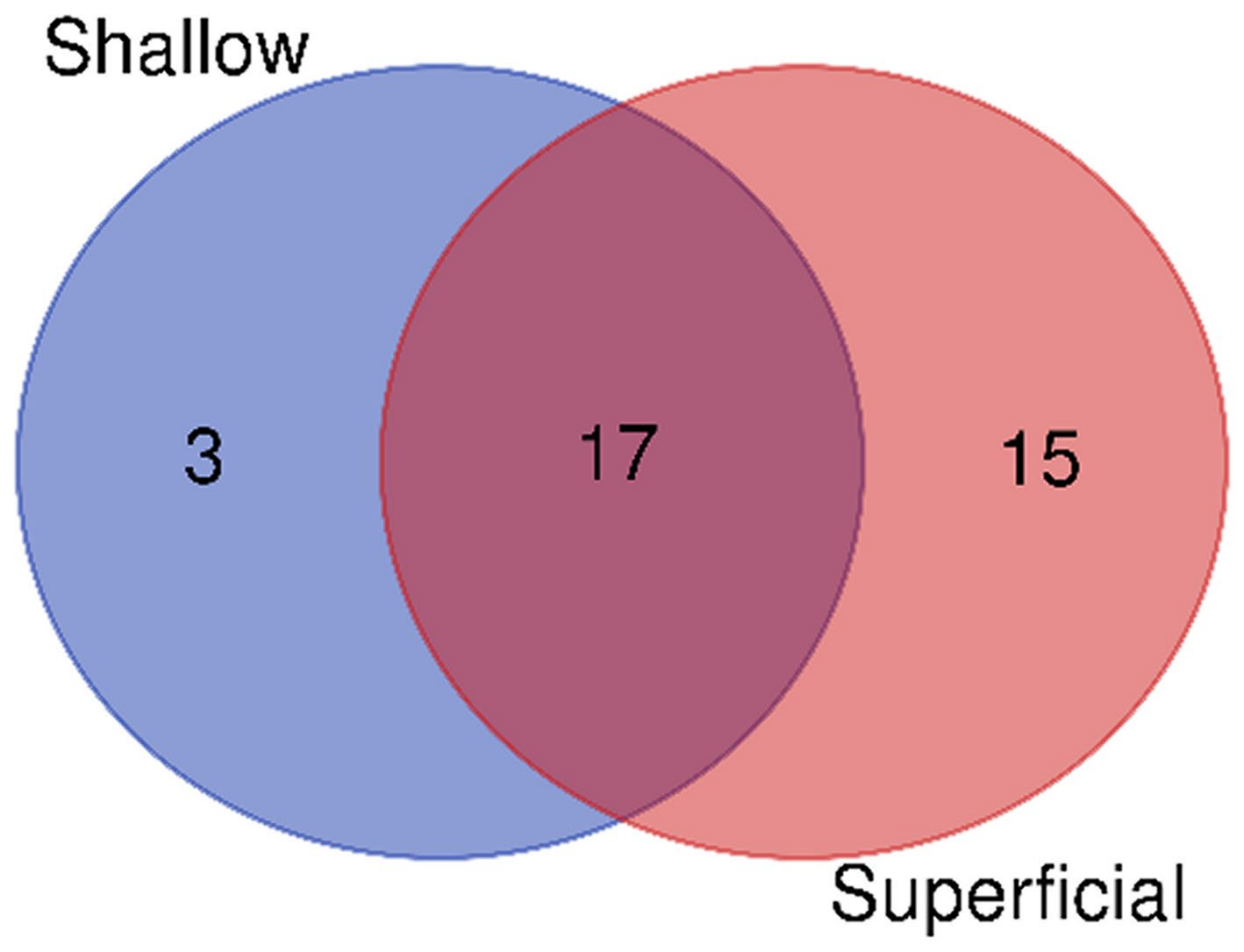
Comparative pathway mapping of virulence factors in different depth environments.

In the superficial ulcer group, virulence factors detected were significantly higher and included the detection of 221 factors from seven bacterial species. The most frequent among the virulence factors detected in these patients were *Pseudomonas aeruginosa* virulence factors, such as mannuronan synthase, alginate biosynthesis protein AlgA, alginate biosynthesis transcriptional regulatory protein AlgB, and GDP-mannose 6-dehydrogenase (GMD) of the same species, associated with biofilm formation and production of polysaccharides and persistence of bacteria. 127 biological processes were identified in the superficial group, of which 101 processes overlapped with those from the shallow group. These included the pyocyanine biosynthetic process, protein secretion by the Type III secretion system, negative regulation of complement activation, regulation of toxin production, capsular polysaccharide biosynthetic process, and bacterial chemotaxis. Moreover, 26 biological processes specific to the superficial group were identified, including biofilm dispersal and iron acquisition and metabolism. Pathway analysis indicated that superficial ulcers share 17 metabolic pathways with shallow ulcers including, but not limited to glycan biosynthesis, GDP-alpha-D-mannose biosynthesis, pyocyanine biosynthesis, salicylate biosynthesis, and antibiotic biosynthesis (Supplementary Table S5; Figures 2, 3).

In the shallow ulcer group, 144 virulence factors were identified, associated with seven bacterial species. Predominant virulence factors were associated with *Pseudomonas aeruginosa* and were similar in profile to the superficial group, featuring biofilm formation and chronic infection-associated factors. The shallow ulcer group demonstrated a wide array of biological processes with a large number of overlapping terms with the superficial group. There was one biological process, however, which was unique to this group: cytotoxin production. Metabolic pathway analysis showed that shallow ulcers share 17 of the pathways of the superficial group, reinforcing similarities in function among them (Supplementary Table S5; Figures 2, 3).

### 3.5 Microbial composition, virulome, and resistome in Libyan diabetic foot ulcer infections

**Figure 4:**
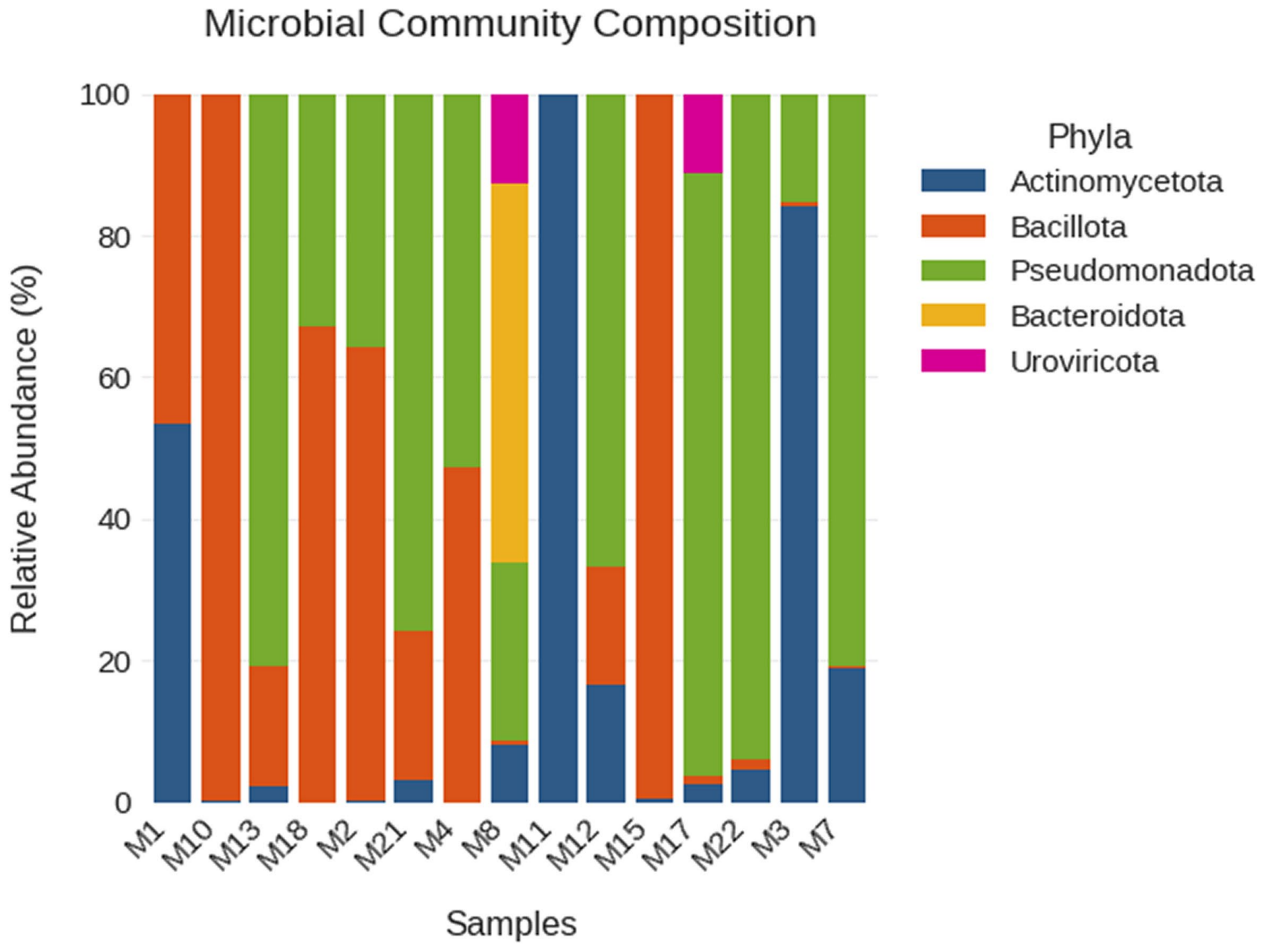
Relative abundance of microbial phyla in Libyan DFU samples.

For this part of the study, a dataset of 12.8 GB of sequencing data was analyzed, obtained from microbiological swabs collected from infected diabetic foot ulcers in 15 Libyan patients. These yielded 43.46 million sequences with a consistent median read length of 149 base pairs. Importantly, all the samples were from clinically infected DFUs; thus, it allows for focused analysis of pathogenic microbial communities associated with infection in wounds. The microbial composition of diabetic foot ulcers in Libyan samples revealed a very complicated and extremely variable taxonomic landscape dominated by four major phyla, namely Actinomycetota, Bacillota, Pseudomonadota, and Bacteroidota. Each sample reflected a different microbial signature, representative of the heterogeneity of such infected wounds (Supplementary Table S5; Figure 4).

Actinomycetota showed great variability between complete absence and complete dominance, with Actinomycetota reached 99.9% abundance in sample M11. Bacillota was truly ubiquitous, occurring in all samples in a wide range of abundance ranging from 0.04 to 99.76%, with samples M10 and M15 almost fully composed of this phylum. Pseudomonadota was also ubiquitously represented across samples, reaching peak abundances as high as 93.90% in samples M4, M13, M17, and M22. In contrast, Bacteroidota remained mostly inconspicuous, except in sample M8, where it comprised 53.58% of the microbial community (Supplementary Table S3). The species-level analysis indicated a high degree of specialization and sample-specificity among the bacterial populations. *Corynebacterium striatum* dominated sample M11 (99.2%), M7 (88.4%), M3 (85.6%), and M1 (83.7%). Sample M12 had insufficient microbial reads for species-level quantification (<5 classified reads total). *Pseudomonas aeruginosa* dominated sample M13 (45.8% of classified microbial reads following CSS normalization) and was the sole species of interest in sample M22, though the latter yielded only 259 microbial reads and should be interpreted with caution. *Staphylococcus aureus* was the dominant species in sample M2 (53.0%), co-occurring with *Acinetobacter johnsonii* (33.2%). *Enterococcus faecalis* was dominant in M18 (64.4%) and M15 (59.8%), and co-dominant with *Staphylococcus aureus* in M10 (*E. faecalis* 49.1%, *S. aureus* 46.4%).

Among them, *Acinetobacter baumannii* dominated sample M21 (53.3%). Sample M8 was characterized by a polymicrobial community dominated by *Myroides profundi* (21.7%) and *Myroides odoratimimus* (18.4%) alongside *Corynebacterium striatum* (16.1%), *Staphylococcus aureus* was dominant in M2 (53.0%) and co-dominant in M10 (46.4%). Sample M3 was dominated by *Corynebacterium striatum* (85.6%). Other less predominant species (including *Proteus mirabilis*, *Stenotrophomonas maltophilia*, and *Cutibacterium acnes*) happened throughout but had significantly fewer reads and highly dispersed features of representation in those samples (Supplementary Tables S3, S5). Virulence factor annotation and resistome profiling across the Libyan cohort were performed on all 15 samples. Detection capacity varied substantially across samples as a direct function of available microbial sequencing depth (Supplementary Figure 3, 8; Supplementary Table S2). Samples M13 and M2 yielded sufficient microbial reads for assembly-based virulome and resistome analysis (1,431,381 and 291,436 microbial reads, respectively); the remaining 13 samples had high host DNA content (mean 89.3%, range 73.8–98.5%), resulting in limited microbial assembly depth. The absence of detected virulence factors or ARGs in these 13 samples reflects insufficient microbial sequencing depth for annotation, not biological absence of these determinants.

### 3.6 Alpha diversity comparison between infected DFU samples from Libya and non-infected DFU samples from USA public data

Shannon’s diversity index for the US reference cohort ranged from 0.92 (sample 312) to 4.415 (samples 126 and 153), with a mean of 2.83 ± 0.85 (*n* = 25). The Libyan characterization cohort yielded Shannon indices ranging from 0.0 (M12) to 1.293 (M13) (mean 0.474 ± 0.399, *n* = 15). Mann–Whitney U test indicated a significant difference (*U* = 375, *p* < 0.0001); however, this observation must be interpreted with caution. The two cohorts differ simultaneously in infection status, geographic origin, bacterial burden, library preparation, and sequencing pipeline, multiple co-varying factors any one of which could contribute to the observed diversity difference. This between-cohort comparison is therefore presented as descriptive and hypothesis-generating only; it cannot be causally attributed to infection status. This observation is consistent with the known ecological principle that clinically infected wounds exhibit reduced microbial diversity relative to non-infected wounds (Pouget et al., 2020; Mudrik-Zohar et al., 2022; Kalan et al., 2019), but validation using matched infected/non-infected patients from the same North African cohort would be required to confirm this causal relationship. Following rarefaction to equal microbial read depth (55,000 reads), the diversity difference was maintained for samples with sufficient microbial coverage (Mann–Whitney *U* = 300, *p* < 0.0001), confirming that sequencing depth differences do not explain the observed pattern. The CARD database was employed in the investigation of the AMR profiles of microbial communities in DFU samples from Libyan patients. There was significant variation in the resistome between the samples. Sample M13 had the highest diversity of resistance genes, with 61 detected, while Samples M2, M10, and M15 were characterized by a lower number of AMR determinants. On the other hand, no detection of AMR genes from the rest of the samples evidenced a lower resistance profile in these cases (Supplementary Table S5). In this study, some of the resistance genes identified in M13 included those conferring resistance to a wide range of antibiotics: aminoglycosides (gentamicin and kanamycin), macrolides, fluoroquinolones, beta-lactams, carbapenems, and tetracyclines. Resistance to these drugs was mediated by genes such as AAC(6′)-Ib4, ANT(4′)-IIb, APH(3′)-Ia, ErmA, ErmB, MexA, MexB, and OXA-50. The presence of MexC, MexD, and other Mex efflux pump genes suggested resistance to a wide range of classes of antibiotics, whereas OXA-1 and OXA-14 were associated with beta-lactam resistance. Besides these, genes such as Tet(G), FosB, and qacH contributed to tetracycline, fosfomycin, and quaternary ammonium compound resistance, respectively (Supplementary Table S5). On the other hand, Sample M10 had a much narrower resistance profile, with only one detected gene, PC1_beta-lactamase_(blaZ), which is resistant to beta-lactams mainly penicillins, hence a rather limited resistance profile in comparison with M13. Sample M15 contained one detected gene, ErmC, which conferred resistance to the antibiotic classes of macrolides, lincosamides, and streptogramin B, such as erythromycin and clindamycin. Sample M2 presented an average resistance profile, in which six ARGs were found: PC1_beta-lactamase_(blaZ) is responsible for beta-lactams resistance. SAT-4, aad(6), and aadA6 confer resistance against aminoglycosides. Also, Tet(G) presents resistance to tetracyclines, while TriC class belongs to a kind of unknown class of antibiotic. M2 also showed the multi-resistant pattern, but this one was not so extended when compared with that obtained from sample M13.

### 3.7 Virulome analysis

The virulome analysis displayed significant variability between the samples; M13 and M2 were the only two samples that had virulence factors. M13 contained a very large array of 225 virulence factors, Of these 225, a subset of 220 were found to be shared with the closest reference strain PSA10 (see Section 3.8), which could only be indicative of a strain carrying a very good amount of pathogenic potential, while M2 had 21, indicating less virulence. The remaining samples did not display any virulence factors (Supplementary Table S5). The absence of detected virulence factors in 13/15 samples reflects technical limitations rather than biological absence. As a result, the virulome and resistome characterization of the Libyan cohort is substantially derived from samples M13 and M2, and generalization to the broader infected DFU population should be made with caution. Analysis of MultiQC quality control data revealed that these 13 samples had 73.8–98.5% host DNA (mean 89.3%), leaving insufficient microbial sequencing depth for VFDB annotation or contig assembly. This pattern is directly supported by the strong negative correlation between host DNA content and virulence factor detection across all 15 samples (Pearson r = −0.95, *p* < 0.0001; Supplementary Figure 9). Samples M12 and M22 (each 98.5% human DNA, <2,000 microbial reads) produced no assemblable contigs. These observations are consistent with published challenges in clinical wound metagenomics, where high host DNA content without prior physical depletion severely limits microbial analysis depth. All virulence factor annotations represent DNA-level genomic potential; active expression was not assessed.

In Sample M13, the detected VFs involved *Pseudomonas aeruginosa PAO1*, which is a Gram-negative opportunistic pathogen adaptable to various means of resistance mechanisms. These fell into several functional categories indicative of the capability to establish infection, evade the host immune system, and persist under hostile conditions. The major virulence factors found in M13 included Type IV pili-pilF, pilT, pilU, pilG, pilH, pilI, which are responsible for twitching motility, biofilm formation, and adhesion to host cells. The biosynthesis genes of alginate were also present, like algB, algW, mucE, and mucP, which give resistance against the host’s immune system through a polysaccharide matrix produced outside. Besides, genes contributing to the biosynthesis of LPS, for example, waaA, waaP, waaG, gave further support to structural integrity and its capability of immune evasion (Supplementary Table S5). Quorum sensing genes are also detected; rhlI and lasI, suggesting there is a potential for the coordination of virulence gene expression with bacterial population density. The other key factors were rhamnolipid biosynthesis genes (rhlA, rhlB, rhlC) involved in the process of biofilm development; several secretion systems, such as Type III (exoY, pscO, pscN, etc.), Type VI (tse3, clpV1, hsiH1, etc.), and flagella-related genes (motY, motB, motA, flgB, etc.), important in host cell manipulation, bacterial competition, and motility. The existence of iron acquisition systems such as pyoverdine biosynthesis (pvdL, pvdG, pvdS, etc.) and pyochelin biosynthesis (fptA, pchI, pchH, etc.) further supported the ability of the strain under iron-limiting growth. Other virulence factors detected included phospholipase C (plcH), exotoxin A (toxA), elastase (lasB), alkaline protease (aprA), and pyocyanin synthesis genes (phzM, phzS); these further underlined the pathogenic potential of M13.

In contrast, Sample M2 had virulence factors associated with *Staphylococcus aureus*, a Gram-positive pathogen that causes a wide variety of infections. The most remarkable virulence determinants included the staphylococcal enterotoxins (sed, sea) highly powerful superantigens implicated in food poisoning and toxic shock syndrome. Also, genes contributing to intercellular adhesion were present: icaR, icaA, icaD, icaB, which point to the ability of biofilm formation and resistance to immune clearance. Capsule biosynthesis genes were identified, such as cap8F, cap8G, cap8L, and cap8O, which play a protective role against host immune responses, especially phagocytosis. Hemolysins, including hld, hlb, and hly/hla, were present, which take part in the destruction of host tissues by lysing red blood cells. Besides, the presence of Type VII secretion system genes, such as esxA, esaC, esxB, and esaB, indicated its involvement in the exportation of virulence factors for better survival and persistence. The other virulence-associated genes were: staphylokinase, sak, which facilitated bacterial spread by clot lysis; and the proteases, sspA and sspC, that degrade the host proteins and thus support immune evasion (Supplementary Table S5).

### 3.8 Genomic quality assessment, taxonomic classification, and antibiotic resistance profile of the MAG

**Figure 5:**
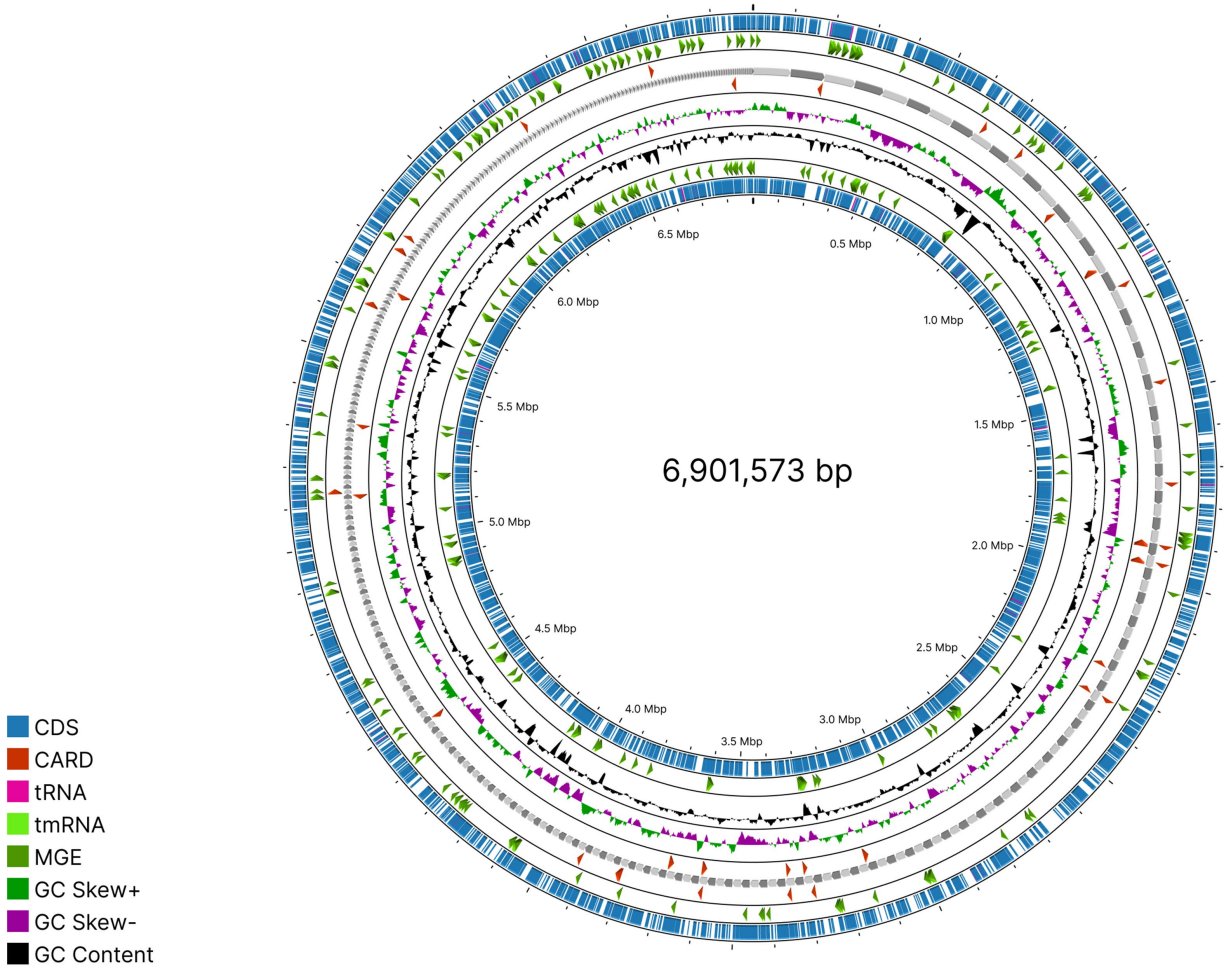
Circular genome map of the MAG.

Among the two samples analyzed (M13 and M2), only one MAG had passed the Minimum Information about a Metagenome-Assembled Genome criteria from sample M13 (Figure 5). CheckM analysis confirmed this by showing high quality with 99.68% completeness and 0.89% contamination. These metrics satisfy MIMAG HIGH quality standards (completeness ≥90%, contamination <5% (Alneberg et al., 2014)).

MAG M2, assembled from sample M2, did not meet MIMAG thresholds (completeness 57.26%, contamination 0.49%) and was therefore excluded from MAG-based analyses; all MAG-derived findings reported in this manuscript pertain exclusively to MAG M13. To confirm the absence of assembly-based false positives in ARG detection, all 60 CARD-identified ARGs in MAG M13 were independently validated using RGI v6.0.2 (protein homolog model, strict bitscore cutoff) applied directly to unmapped reads from sample M13.

**Figure 6:**
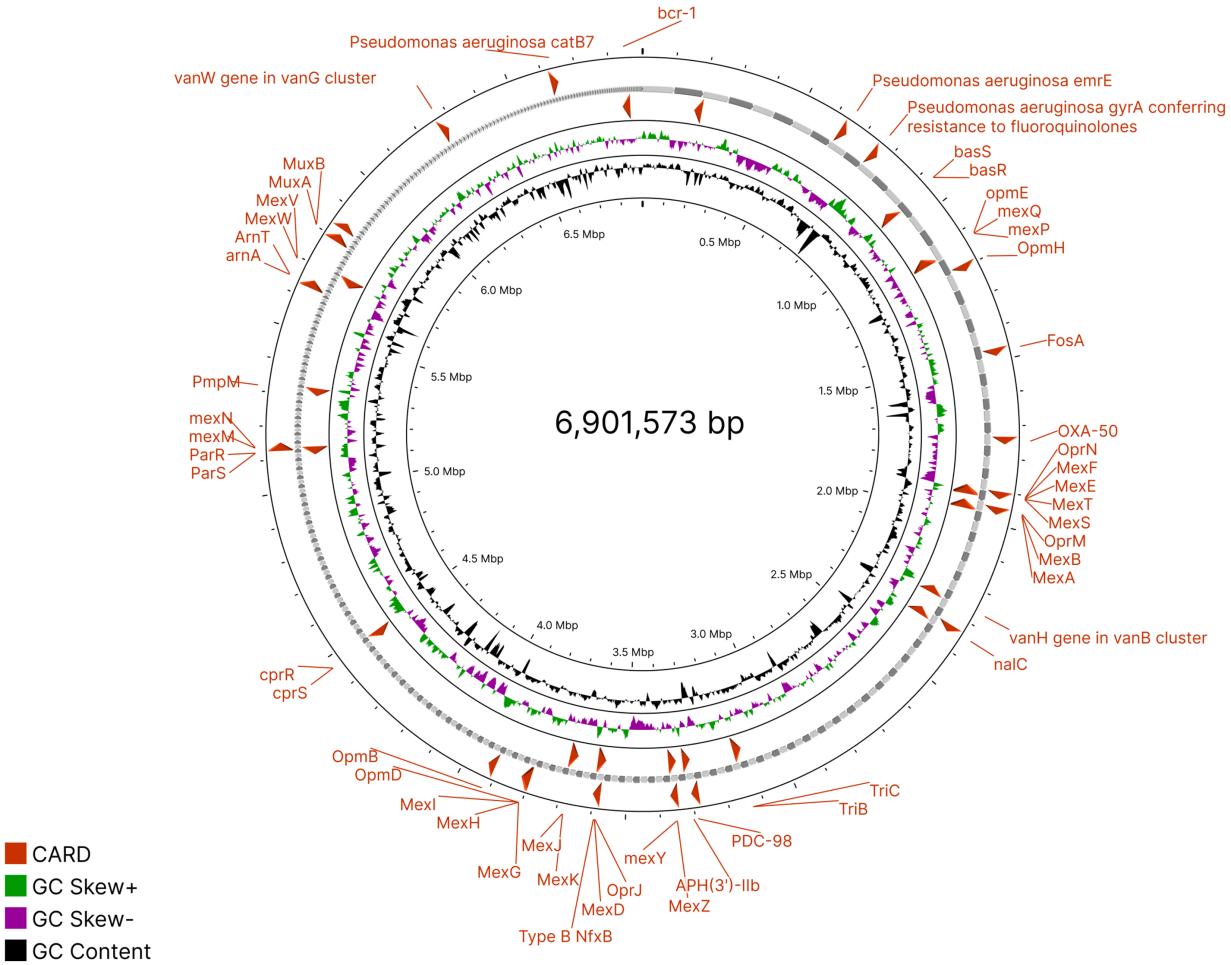
Circular genome map of ARG annotations in the MAG.

Taxonomic classification through the GTDB toolkit assigned this MAG to *Pseudomonas aeruginosa*. FastANI revealed that it shared 99.25% similarity with the reference genome *Pseudomonas aeruginosa strain NCTC10332* (GCF_001457615.1). Phylogenetic placement (pplacer) also agreed with this classification, pointing to this MAG having a consistent taxonomic assignment with its reference genome (Supplementary Table S6). Antibiotic resistance genes from the recovered *Pseudomonas aeruginosa* MAG were identified (Figure 6) and compared with the previously detected ones in the sample source M13 to assess the potential of antimicrobial resistance. It was observed that 60 out of those ARGs identified within the MAG were also present in M13; these confer resistance against diverse classes of antibiotics: aminoglycosides, beta-lactams, fluoroquinolones, macrolides, and tetracyclines. Among the resistance determinants identified, APH(3′)-IIb, arnA, FosA, and bcr-1 encode resistance to aminoglycoside and fosfomycin. The Mex efflux pump system includes MexA, MexB, MexC, MexD, MexE, MexF, MexG, MexH, MexI, MexJ, MexK, MexL, mexM, mexN, mexP, mexQ, MexV, MexW, and mexY-mediate resistance against various drugs via the export of antibiotics out of the bacterial cell. OprM, OprJ, OprN, OXA-50, and PmpM were detected, which encode further resistance against beta-lactams and carbapenems (Supplementary Table S6). Importantly, ARGs, including emrE and soxR, were found in *Pseudomonas aeruginosa*-specific resistance mechanisms and thus gave relevance to conserved mechanisms of resistance among this species. Additionally, TriA, TriB, and TriC established further the capability of this strain to resist the antimicrobial pressure.

### 3.9 Phylogenetic and comparative genomic analysis of MAG from M13

**Figure 7:**
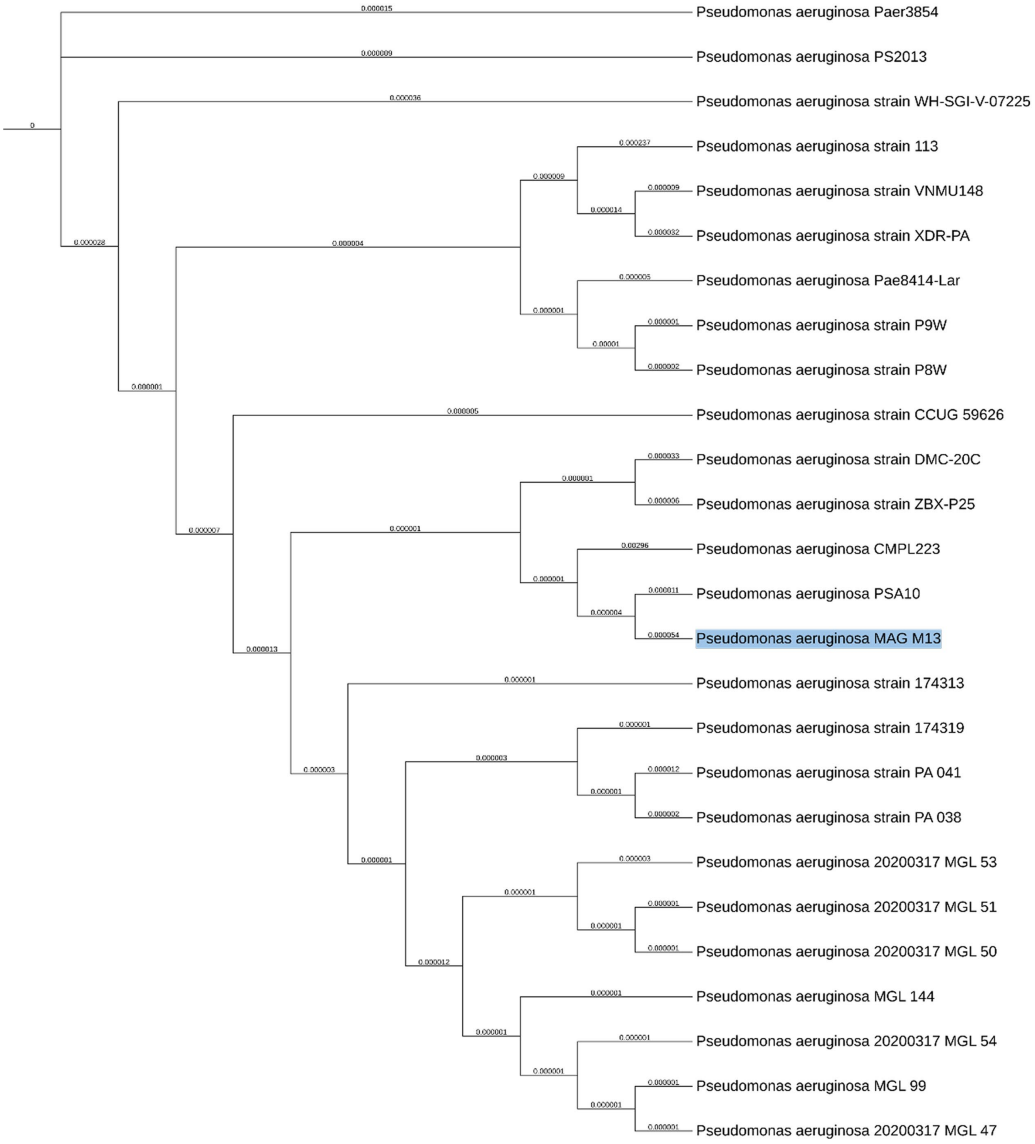
Phylogenetic position of *P. aeruginosa* MAG M13 among related strains.

To validate the taxonomic position of MAG M13, a phylogenetic analysis was performed with strains showing the closest genetic distance and having the same MLST sequence type ST664 (Figure 7). The phylogenetic tree presented MAG M13 clustering tight with *Pseudomonas aeruginosa strains PSA10* and CMPL223, being PSA10 (GCA_032844025.1) is closer relative with a genetic distance of 0.000054. A small branch length is supportive that this is indeed a member of *P. aeruginosa* due to the high degree of similarity (Supplementary Table S6).

**Figure 8:**
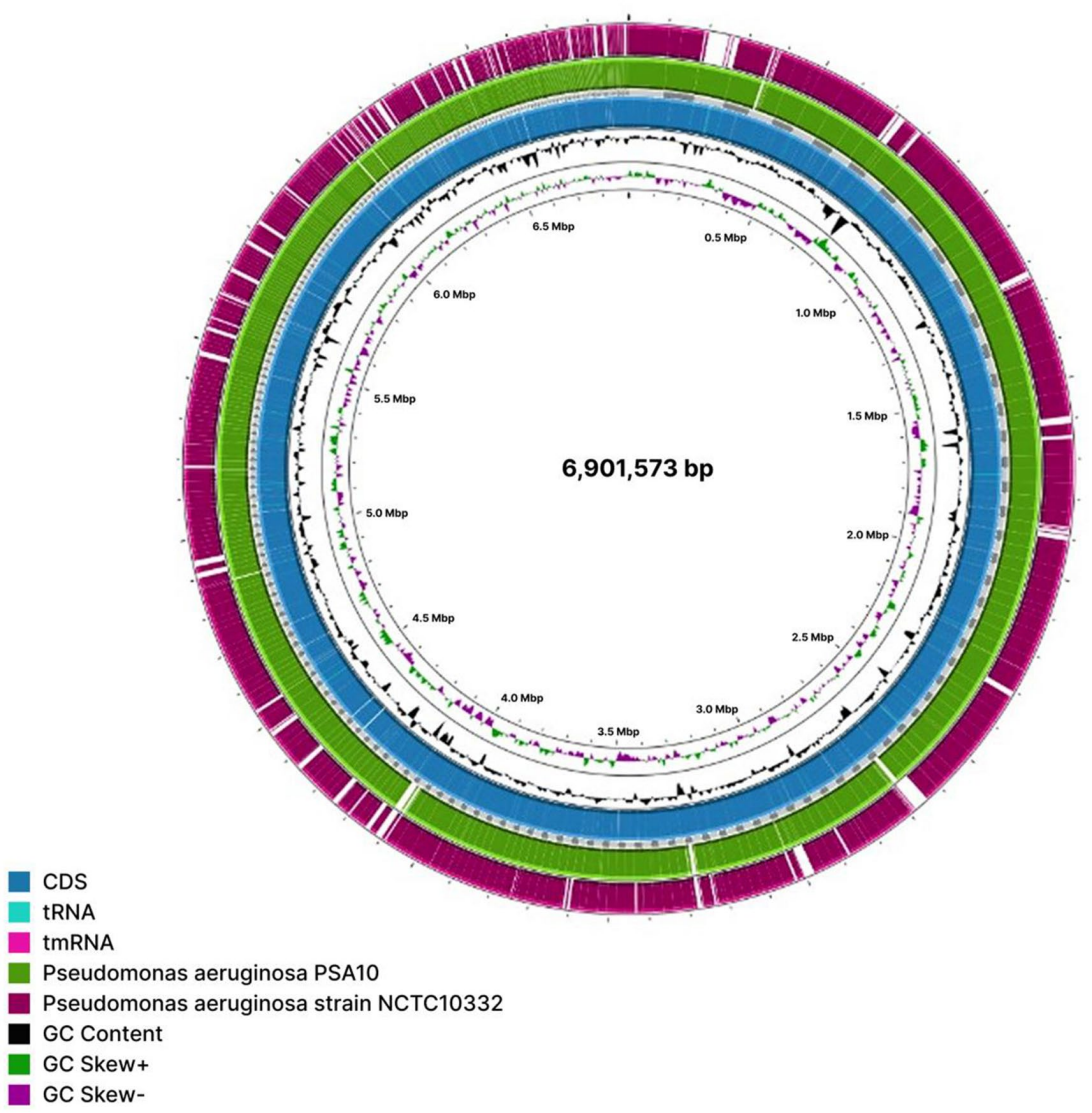
Genome coverage comparison of *P. aeruginosa* MAG M13 against reference strains PSA10 and NCTC10332.

Besides, BLAST analysis against strains PSA10 and *NCTC10332* -the closest reference genome identified by GTDB-, showed that MAG M13 had greater genomic coverage with PSA10, further supporting the phylogenetic results (Figure 8). Analysis of pathogenicity in the closest strain to MAG M13, PSA10, revealed that the MAG and PSA10 shared 220 virulence factors and 60 ARGs in common, thus pointing to genetic and functional similarities to their pathogenic potential (Supplementary Table S6).

### 3.10 Prevalence of mobile genetic elements and genome plasticity in MAG M13

MAG M13 from sample M13 was highly abundant in mobile genetic elements. MobileOG-db detected 213 features related to MGEs, including phage = 78, transfer = 58, replication/recombination/repair = 37, integration/excision = 26, stability/transfer/defense = 14. Accordingly, the very high abundance of the phage-related (32.3%) and transfer-associated features (28.7%) is indicative that HGT might have played an important role in shaping genome structure. Its closest reference strain, PSA10, harbored a nearly identical number of MGEs (472) with a similar distribution pattern (Supplementary Table S6). Besides this, Alien Hunter analysis revealed several genomic regions of atypical nucleotide composition that could represent horizontally acquired genomic islands. These probably correspond to the mobile elements identified by the mobileOG-db analysis and further, reinforce the hypothesis of an extensive genome plasticity in M13 (Supplementary Table S6).

## 4 Discussion

DFUs are a major concern for healthcare systems because these ulcers are chronic and susceptible to infection and are often resistant to antibiotics (Xia et al., 2021). Despite better wound management, DFU treatment is difficult due to the fluctuations of the microorganisms, affecting the nature of infections and treatment efficacies (Doğruel et al., 2022). Growing evidence suggests that the microbiome plays a significant role in resistance to treatment and wound healing; however, the influence of microbial changes remains ill-understood (Canchy et al., 2023). In the present study, shotgun metagenomics and computer-based functional analysis were used to characterize DFU microbiomes at various depths within the ulcer and different stages of infection. This insight sheds new light on how pathogens change to resist antibiotics and may be targeted for treatment. Furthermore, this is the first metagenomic study on DFUs from North Africa, filling a major gap in regional microbiome research. By incorporating data from the nested U. S. group of non-infected DFUs and the Libyan group of infected DFUs, we show how microbes change with depth and infection-status widespread antibiotic resistance, and specific ways pathogens cause harm. This can lead to more precise medical approaches in treating long-lasting wounds.

While microbial richness in the cohort of U. S. non-infected DHFs remained relatively constant across ulcer depths, beta diversity, as expected, clustered significantly by depth, which reflected the microbial community structures dependent on ulcer severity. Deeper ulcers were enriched in fusobacteria, an important phylum associated with biofilm formation and chronic inflammatory conditions (Park et al., 2016). Fusobacteria are indeed the most studied bacteria in the literature on periodontitis and colorectal cancer, where they do not allow immune clearance and thus create localized inflammatory conditions that may interfere with and delay healing in deep DFUs (Muchova et al., 2022). Superficial ulcers had a greater abundance of bacillota, such as Staphylococcus spp. and Enterococcus spp., indicating a greater contribution of environmental exposure rather than deep tissue invasion. These bacteria are known to colonize wounds early and subsequently transition to commensals or opportunistic pathogens depending on wound microenvironment and host immune status (Gjødsbøl et al., 2006; Wolcott et al., 2013). An indication of potentially pathogenic microbial communities even within clinically non-infected wounds emerged from functional analysis of non-infected DFUs aside from taxonomic shifts. Genes responsible for adhesion, biofilm formation, and iron acquisition were detected as being very common, further corroborative evidence that the mechanisms causing the persistence of pathogens arose before frank infection. Deep ulcers were upregulated with oxidative pressure resistance pathways indicating that microbes residing within chronic wounds are adapting to hypoxic, nutrient-deficient environments features of biofilm-based infections. “Quorum sensing regulators” and “extracellular polysaccharide biosynthesis genes” will be identified as target candidates for early-stage biofilm formation; even worse, these wounds show no signs of infection during the clinical examination.

An interesting finding in the U. S. cohort was the presence of antibiotic resistance genes (ARGs) in non-infected DFUs which were mainly superficial ulcers. This supports the viewpoint that these ulcers may even store antimicrobial resistance before the appearance of any clinical infection. While several beta-lactamase and aminoglycoside-modifying enzyme genes were detected, it indicates that early resistance may be in the process of accumulation. The majority of Macrolide efflux pumps were prevalent thereby contributing to the multidrug resistance-MDR phenotype, which may complicate treatment strategies. Higher environmental exposure, or even a previous antibiotic treatment, for superficial ulcers indicates ARG enrichment hence the requirement for metagenomic-guided antimicrobial stewardship to decrease resistance selection (Jouhar et al., 2020). Though alone, the presence of genes does not confirm expression, detection of these resistance determinants points toward the growing requirement for proactive antibiotic resistance surveillance in DFUs, even before any visible clinical signs of infection appear. Such findings have important ramifications for the management of diabetic foot ulcers (DFU) and antimicrobial therapy. Thus, the choice of antimicrobial agents may be tailored according to the depth of the wound, given that microbiome detection of Fusobacteriota in deeper ulcers indicates that anaerobic antimicrobial therapy with metronidazole might also offer an advantage in addition to standard care. The peculiarity of *P. aeruginosa* harboring efflux pumps would require an additional consideration of efflux pump inhibitors as therapeutic adjuncts for DFUs.

In the Libyan characterization cohort, lower Shannon diversity was observed alongside dominance by opportunistic pathogens. While this pattern is consistent with infection-associated community shifts documented in prior studies (Pouget et al., 2020; Mudrik-Zohar et al., 2022), direct causal attribution to infection status is not possible given the multiple co-varying differences between the two cohorts described in the Limitations section. The most predominant taxa include *Pseudomonas aeruginosa*, a leading MDR pathogen in chronic wounds, and a major contributor to biofilm persistence (Garousi et al., 2023); *Staphylococcus aureus*, known for co-infection with *P. aeruginosa*, resulting in worsened clinical outcomes and increased treatment resistance (Hoffman et al., 2006; Hotterbeekx et al., 2017); and *Corynebacterium striatum*, an opportunistic pathogen with increasing incidence in various human infections, is an emerging multidrug-resistant organism, as reported in a previous Tunisian study (Alibi et al., 2017). Commonly resistant to antimicrobial drugs, it is therefore related to the increased use of parenteral antimicrobial agents (Hahn et al., 1908). Functional characterization of infected DFUs revealed a remarkable increase in virulence-associated genes, including proteases, hemolysins, and secretion systems, these being important for degrading host tissues and evading the immune response (Citron et al., 2007; Rajab and Hegazy, 2023). Also abundant were genes associated with biofilm, in agreement with previous reports that microbial virulence in DFUs is often driven by biofilm-mediated persistence (Pouget et al., 2020; Afonso et al., 2021; Mamdoh et al., 2023; Pouget et al., 2021).

Among the infected diabetic foot ulcers (DFUs) sampled from Libyan patients, sample M13 emerged as having an extremely high load of ARG and virulence traits, which demanded metagenome binning to reconstruct high-quality *Pseudomonas aeruginosa* genome (MAG M13). On comparative genomic analysis, this MAG M13 was found to share 99.25% similarity with the *Pseudomonas aeruginosa* isolated PSA10, a highly virulent MDR strain previously reported for a urinary tract infection in Italy (Genbank Accessions: JAWIWQ000000000). The major genomic features included 220 matching virulence factors further confirming increased pathogenicity due to 60 shared ARGs giving resistance to beta-lactams, aminoglycosides, and fluoroquinolones; and several large mobile genetic elements (MGEs) indicating genome plasticity and presenting a strong potential of horizontal gene transfer (HGT). ST664 is an emerging multidrug-resistant clone of *Pseudomonas aeruginosa* that is increasingly found within healthcare settings worldwide. It has already been detected in clinical isolates from burn clinics and hospitals (Diorio-Toth et al., 2022; Vaez et al., 2017; Salem et al., 2024), demonstrating the significance of patient populations that may be highly susceptible to infection by this emerging clone. Hospital wastewater has also been implicated in the isolation of the strain (Khan et al., 2022), reaffirming the environmental fate inside medical facilities. Our research presented the detection of ST664 from diabetic foot ulcers DFUs, describing a highly resistant and virulent isolate that could potentially emerge as a threat to chronic wound infections. Since ST664 was found in DFUs, it gives rise to speculation concerning the possibility of accumulation of antibiotic resistance gene ARG and bacterial adaptation through horizontal gene transfer HGT, which would make treatment difficult. The proliferation and distribution of ST664 in various medical settings, including DFUs, underscore the need for proper surveillance and rigorous infection control mechanisms to limit its spread and impact on patients’ outcomes. Future research should examine if the environment of DFU favors ARG accumulation and promotes adaptability among bacteria, rendering therapy more difficult.

Several limitations of this study must be acknowledged explicitly. First, the two cohorts compared, the US non-infected reference dataset and the Libyan infected characterization cohort, differ simultaneously in infection status, geographic origin, host ethnicity, healthcare system, antibiotic exposure history, wound depth distribution, sampling technique (swab post-debridement), DNA extraction protocol, and sequencing platform. These co-varying differences preclude causal attribution of any observed microbiome differences to infection status alone; all between-cohort observations are descriptive and hypothesis-generating. Second, microbial sequencing depth in the Libyan cohort varied substantially across samples (0–1,431,381 microbial reads) due to inter-patient differences in bacterial burden at time of swabbing, a biologically informative finding but one that constrained the analytical depth achievable in lower-burden samples. Thirteen of 15 samples had insufficient microbial depth for virulome or resistome annotation; this is a recognized challenge in clinical wound metagenomics using swab sampling without prior physical host depletion (Mudrik-Zohar et al., 2022; Morsli et al., 2024; Kalan et al., 2019). Third, all VF and ARG annotations represent genomic resistance potential at the DNA level; phenotypic resistance was not confirmed by MIC testing. Fourth, the absence of matched non-infected Libyan controls is an acknowledged limitation; future studies should recruit concurrent non-infected Libyan DFU patients under identical protocols to enable properly controlled comparisons.

DFUs now face the bitter threat of multidrug resistance (MDR), which impels alternative forms of therapy to be sought. Phage therapy had indicated efficiency in the preclinical tests in selectively targeting *P. aeruginosa* and *S. aureus* with bacteriophages and offered a precision approach to involve other high-antibiotic resistance gene (ARG) DFUs (Gomez-Ochoa et al., 2022; Danis-Wlodarczyk et al., 2016). Also, biofilm-disrupting agents such as DNase and several quorum-sensing inhibitors are likely to facilitate antibiotic diffusion, thus raising treatment benefits (Mishra et al., 2020). Shotgun metagenomics will be included in clinical practice for rapid detection of ARGs and personalized treatment with less reliance on broad-spectrum antibiotics (Sanabria et al., 2021); this culture-independent diagnostics approach will allow strain-level resolution of pathogens from biofilms and anaerobic bacteria that culture methods may miss. Such combinations would enhance pathogen identification and improve the precision of treatments for DFUs. A complete metagenomic study of DFU microbiomes that reports on microbial shifts with depth, broad antibiotic resistance, and virulence mechanisms specific to certain pathogens has been provided in this work. By fusing genomic, functional predictive, and resistance profiling approaches, our findings provide an avenue for the potential use of precision medicine in DFU treatment. The next step is to carry out longitudinal tracking of the DFU microbiome, assessment of host–microbe interactions, and targeted therapies, which could be cardinal in ameliorating chronic wound management and tackling multidrug-resistant infections in DFUs across the globe.

## 5 Conclusion

This first shotgun metagenomic report on DFUs from North Africa provides two primary contributions: depth-stratified ARG ecology in non-infected DFUs from the US reference cohort, and the first genomic characterization of a hypervirulent *P. aeruginosa* ST664 strain from a Libyan infected DFU. The US reference cohort revealed depth-dependent microbial stratification with clinically significant ARGs including mecA and the mexAB-oprM efflux system, present in non-infected wounds, underscoring the need for proactive resistance surveillance. The Libyan characterization cohort identified four dominant opportunistic pathogens (*P. aeruginosa, S. aureus, A. baumannii, C. striatum*) with high inter-patient heterogeneity. MAG reconstruction from sample M13 yielded a high-quality *P. aeruginosa* ST664 genome (99.68% completeness, 0.89% contamination) carrying 220 virulence factors, 60 ARGs, and 213 mobile genetic elements, the first characterization of this emerging MDR clone in a North African DFU and the first shotgun metagenomics data from Libya.

## Data Availability

The datasets presented in this study can be found in online repositories. The names of the repository/repositories and accession number(s) can be found in the article/Supplementary material.

## Footnotes

1 https://www.microbiomeanalyst.ca/MicrobiomeAnalyst/home.xhtml, Accessed December 10–11, 2024.

2
https://github.com/tseemann/shovill

3
https://github.com/tseemann/abricate

4 http://bioinformatics.psb.ugent.be/webtools/Venn/, Accessed December 18–20, 2024.
