# Supplementary material for: Metagenomic characterization of infected diabetic foot ulcers in North Africa: microbial diversity, virulome, and resistome profiling
Source: Front Microbiol. 2026 May 20;17:1825173. doi: 10.3389/fmicb.2026.1825173 (PMC13230127; doi:10.3389/fmicb.2026.1825173)
Supplement: Supplementary file 1 [file Data_Sheet_1.pdf]

Supplementary Material

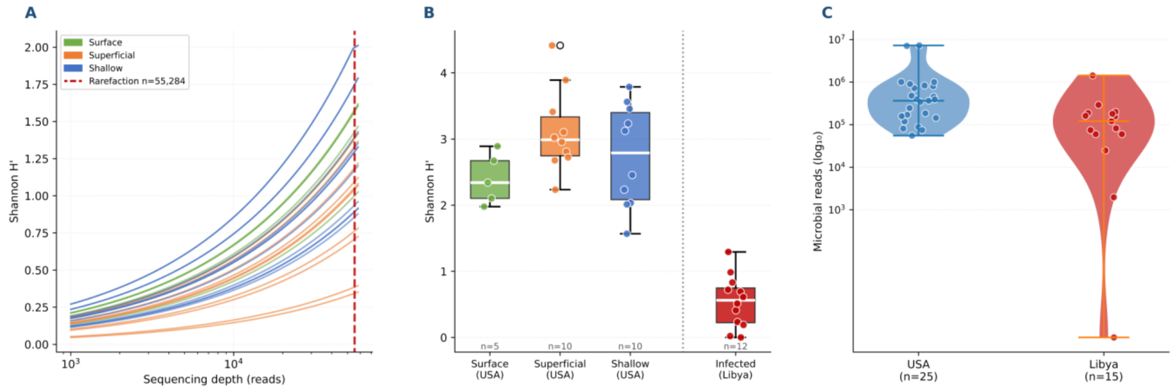

Supplementary Figure 1. Rarefaction analysis: USA discovery cohort. (A) Rarefaction curves for all 25 samples; dashed red line = rarefaction threshold (n=55,284 reads). (B) Post-rarefaction Shannon H' by depth group and cohort. (C) Microbial read depth distributions (violin plots); cross-cohort comparison is descriptive only

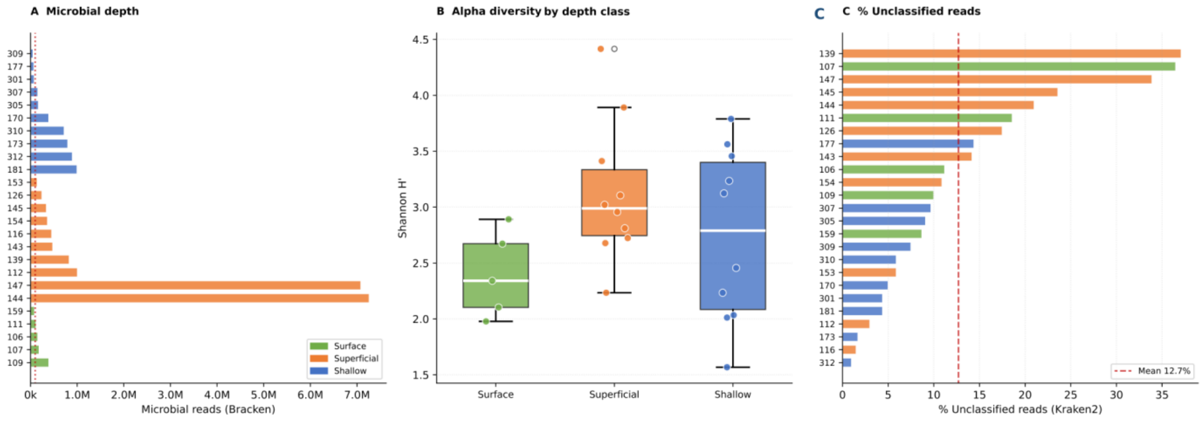

Supplementary Figure 2. Quality metrics and diversity analysis: USA discovery cohort (n=25). (A) Per-sample microbial read depth by ulcer depth class. (B) Shannon H' by depth group; one-way ANOVA F=2.140, p=0.141 (not significant). (C) Percentage of unclassified Kraken2 reads per sample (mean 12.7%)

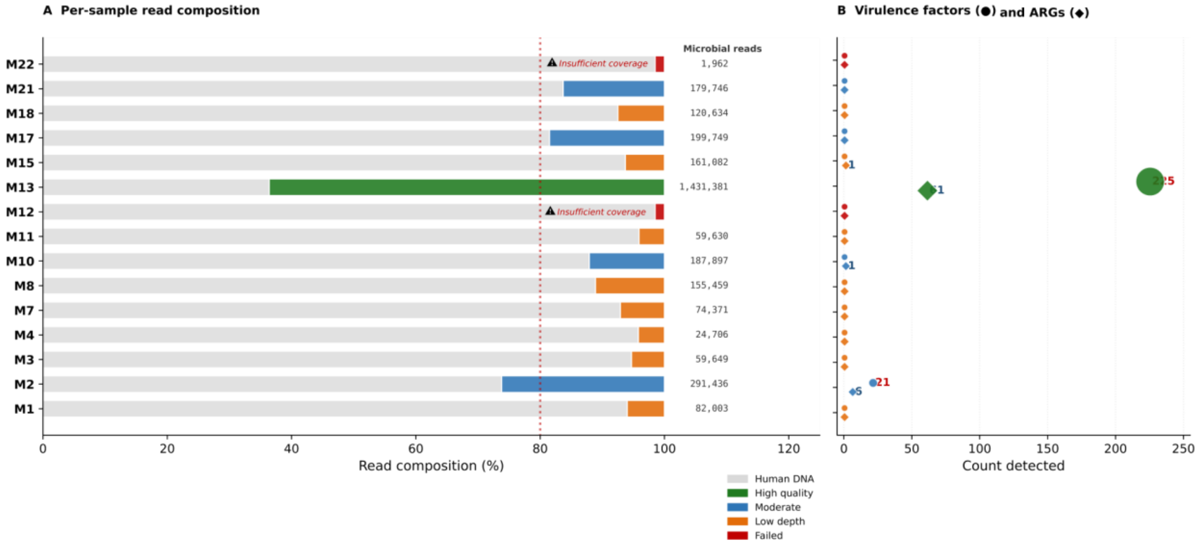

Supplementary Figure 3. Per-sample sequencing quality: Libyan characterization cohort (n=15). (A) Stacked horizontal bars showing human (grey) versus microbial (coloured) read composition; numbers indicate total microbial reads. □ marks samples with insufficient microbial coverage (<2,000 reads). (B) Virulence factors (circles) and ARGs (diamonds) detected per sample

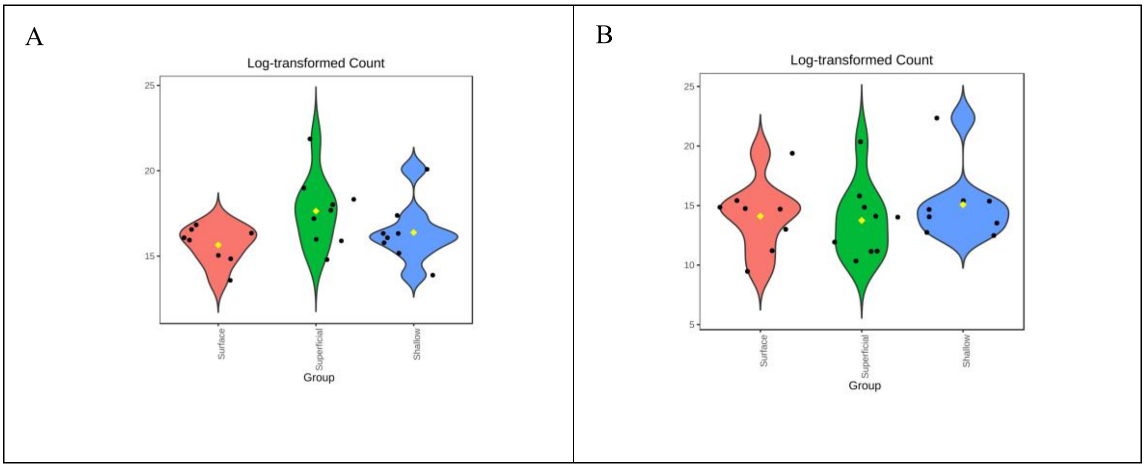

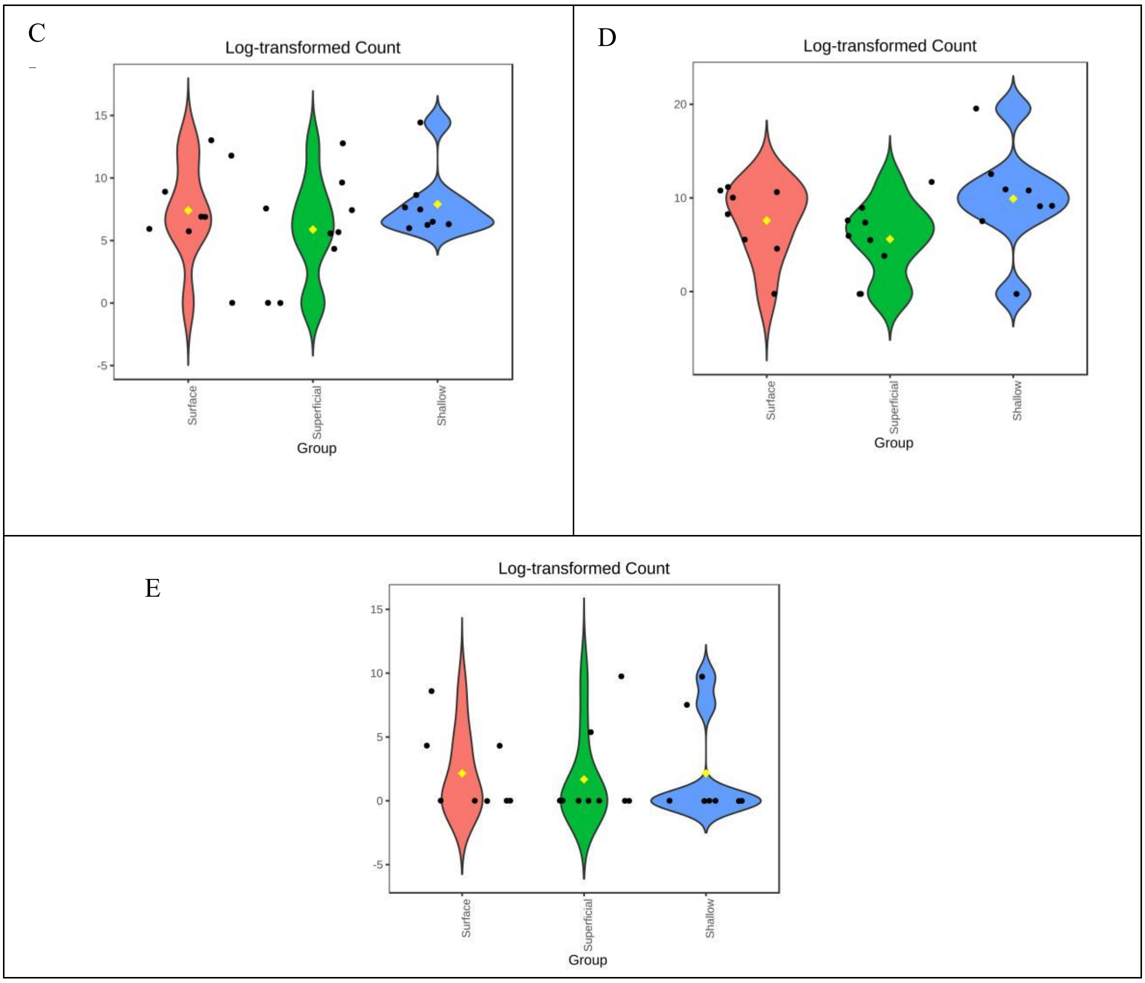

Supplementary Figure 4. : Differential Abundance Across Ulcer Depths (Phylum-Level Distribution). A) Bacillota, B) Bacteroidota, C) Campylobacterota, D) Fusobacteriota, E) Spirochaetota

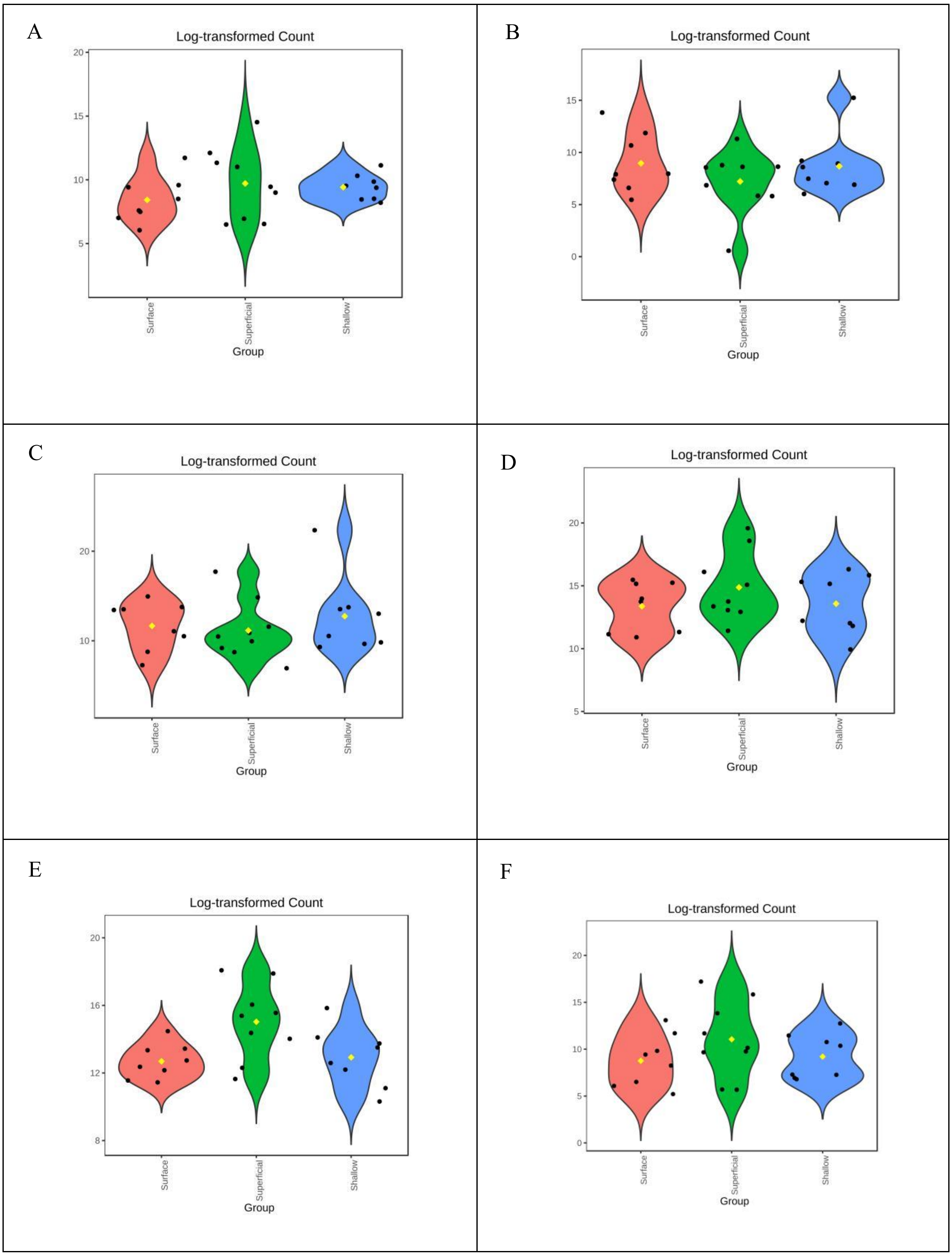

Supplementary Figure 5.: Differential Abundance Across Ulcer Depths (Family-Level Distribution) A) Enterococcaceae, B) Neisseriaceae, C) Prevotellaceae, D) Pseudomonadaceae, E) Staphylococcaceae, F) Veillonellaceae.

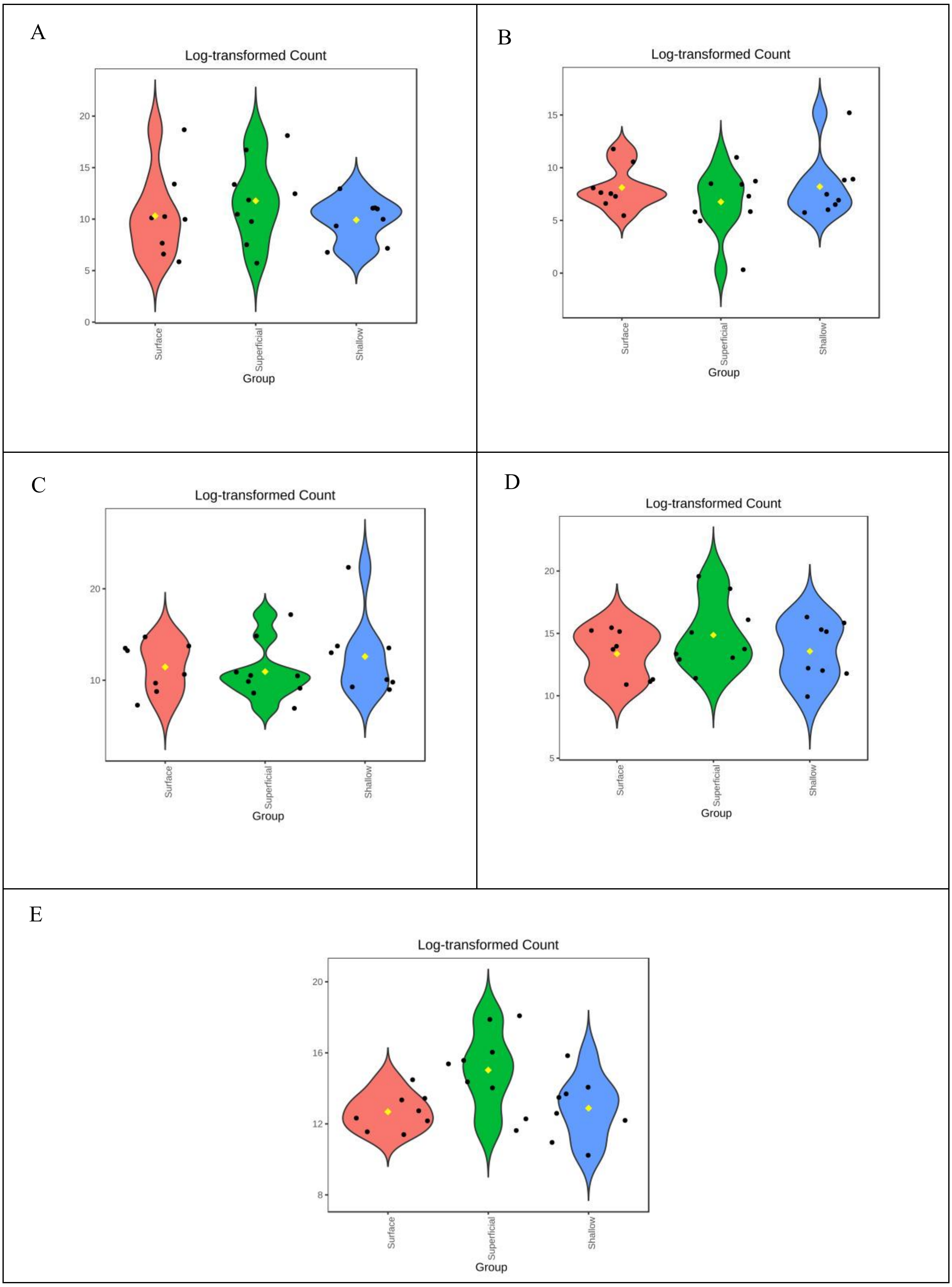

Supplementary Figure 6.: Differential Abundance Across Ulcer Depths (Genus-Level Distribution) A) Haemophilus, B) Neisseria, C) Prevotella, D) Pseudomonas, E) Staphylococcus

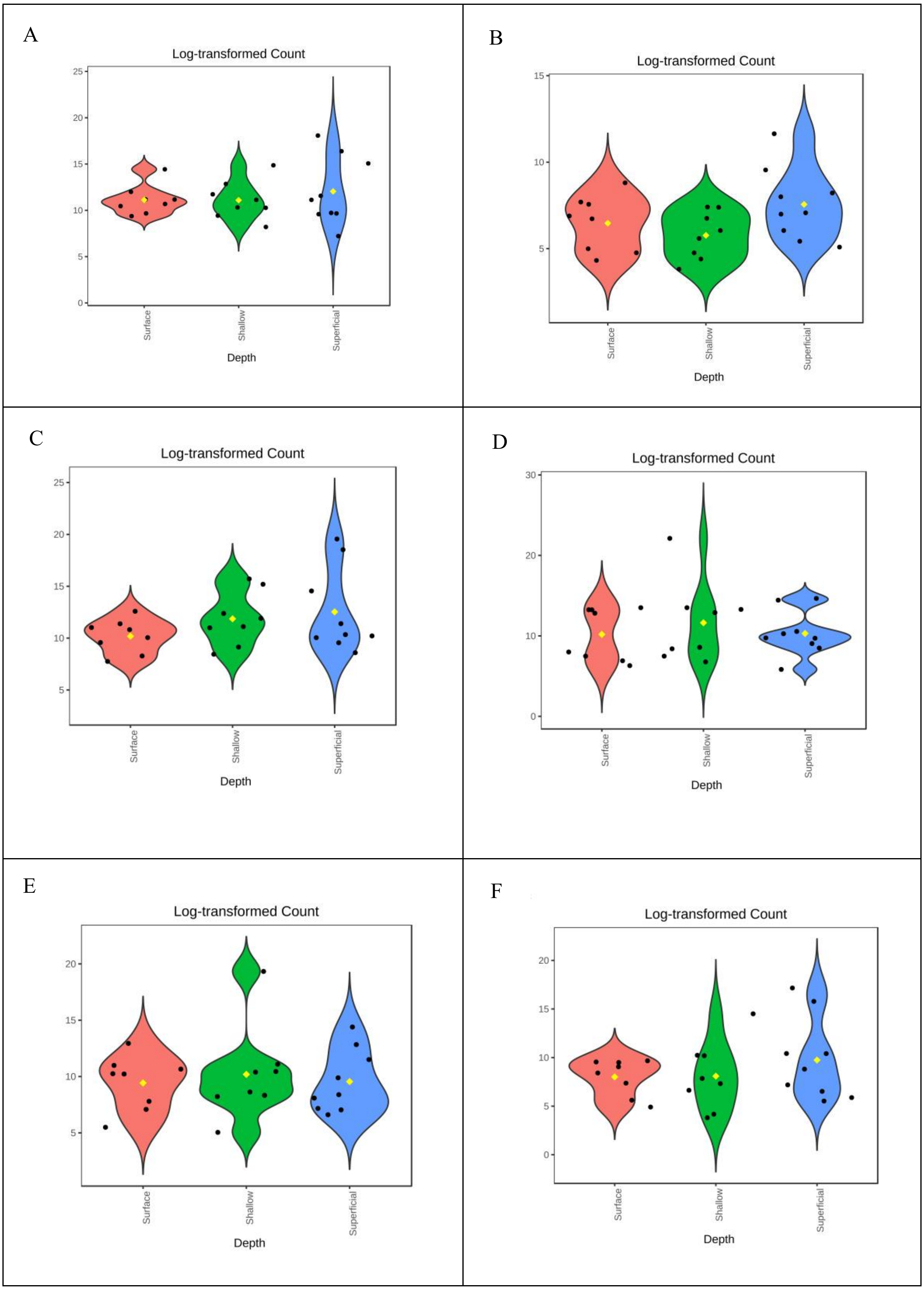

A
Log-transformed Count
0
5
10
15
20
25
Surface
Shallow
Superficial
Depth
B
Log-transformed Count
5
10
15
Surface
Shallow
Superficial
Depth
C
Log-transformed Count
5
10
15
20
25
Surface
Shallow
Superficial
Depth
D
Log-transformed Count
0
10
20
30
Surface
Shallow
Superficial
Depth
E
Log-transformed Count
5
10
15
20
Surface
Shallow
Superficial
Depth
F
Log-transformed Count
0
5
10
15
20
Surface
Shallow
Superficial
Depth

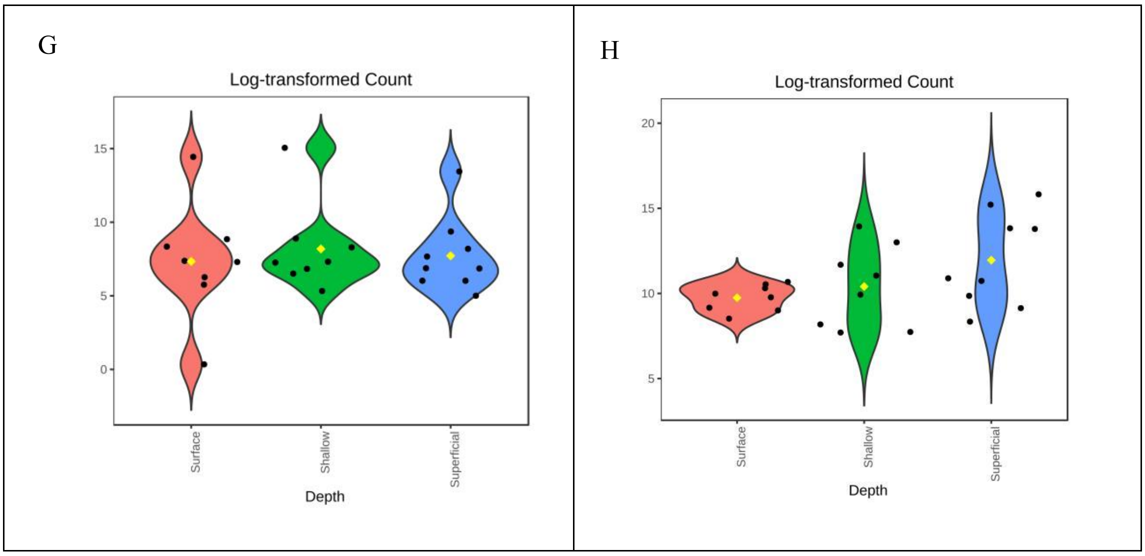

Supplementary Figure 7.: Differential Abundance Across Ulcer Depths (Species-Level Distribution) A) *Staphylococcus aureus,* B) *Rothia mucilaginosa,* C) *Pseudomonas aeruginosa,* D) *Prevotella melaninogenica,* E) *Streptococcus oralis,* F) *Streptococcus mitis,* G) *Streptococcus anginosus,* H) *Staphylococcus epidermidis.*

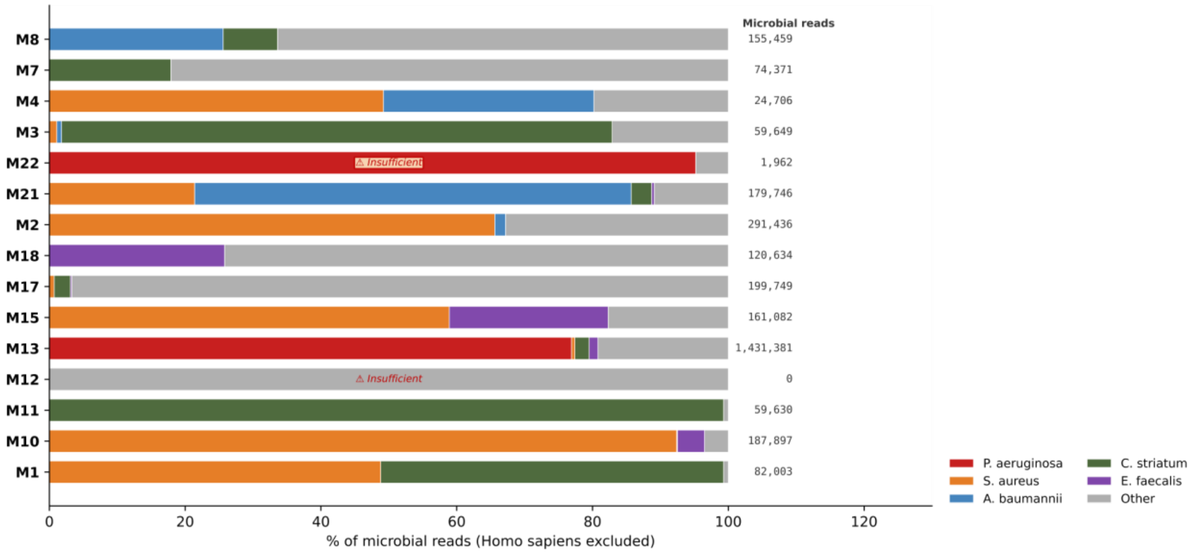

Supplementary Figure 8. Microbial community structure: Libyan infected DFU cohort. Microbial read composition per sample (Homo sapiens excluded); samples with insufficient coverage are indicated.

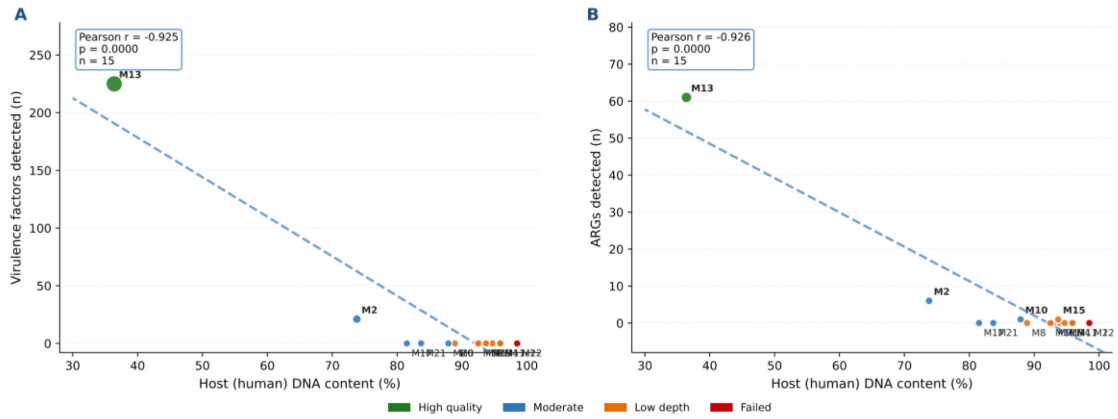

Supplementary Figure 9. Figure R4. Host DNA content predicts genomic detection capacity in Libyan DFU samples. Scatter plots of host DNA percentage versus (A) virulence factors detected and (B) ARGs detected across 15 Libyan samples. Pearson r = −0.95 (VFs) and −0.88 (ARGs); both $p<0.0001$. Colour indicates quality tier (green=high, blue=moderate, orange=low depth, red=failed).
